# Multidimensional Generalized Partial Preference Model for Forced-Choice Items

**DOI:** 10.1017/psy.2025.10054

**Published:** 2025-11-13

**Authors:** Daniel C. Furr, Jianbin Fu

**Affiliations:** 1 Transfr, USA; 2 Educational Testing Service, USA

**Keywords:** dominance model, forced-choice questionnaire, item response theory model

## Abstract

A ranking pattern approach is proposed to build item response theory (IRT) models for forced-choice (FC) items. This new approach is an addition to the two existing approaches, sequential selection and Thurstone’s law of pairwise comparison. A new dominance IRT model, the multidimensional generalized partial preference model (MGPPM), is proposed for FC items with any number (greater than 1) of statements. The maximum marginal likelihood estimation using an expectation-maximization algorithm (MML-EM) and Markov chain Monte Carlo (MCMC) estimation are developed. A simulation study is conducted to show satisfactory parameter recovery on triplet and tetrad data. The relationships between the newly proposed approach/model and the existing approaches/models are described, and the MGPPM, Thurstonian IRT (TIRT) model, and Triplet-2PLM are compared when applied to simulated and real triplet data. The new approach offers more flexible IRT modeling than the other two approaches under different assumptions, and the MGPPM is more statistically elegant than the TIRT and Triple-2PLM.

## 1 Introduction

Noncognitive assessments measure individuals’ attitudes, values, beliefs, or behaviors, and the Likert item format is the most widely used item format for these assessments. The forced-choice (FC) item format (Sisson, 1948) is a popular alternative item format that may mitigate the biases and distortions caused by the Likert item format (e.g., central or extreme tendency, acquiescence, socially desirable responding, and faking; Cao & Drasgow, 2019; Christiansen et al., 2005; Li et al., 2025; Wetzel et al., 2021).

A forced-choice item is a block of two or more statements measuring a common trait or different traits, and test takers are asked to rank the statements within a block based on how well the statements describe their beliefs or behaviors. Various item response theory (IRT) models have been proposed to calibrate FC items and recover normative scores from forced-choice questionnaires (FCQs; sets of forced-choice items) (Brown, 2016, Table 1; Zhang, Tu, et al., 2024, Table 1). All these models assume a judgment on a statement independent of any other statement (i.e., context-free) in an FC item given the latent traits measured by these statements and build the probability models on the “utility” (i.e., psychological value; the term was first used by Thurstone [1929]) of each statement in an FC item. All existing models can be distinguished by two underlying modeling approaches: sequential selection and Thurstone’s law of pairwise comparison.

As we will show later, the existing models have some limitations for FC items with more than two statements. Although statistically sound, the models following the sequential selection approach appear cumbersome, which may make model estimation more challenging. Meanwhile, the common estimation methods for models under Thurstone’s law of pairwise comparison approach are the limited information methods (Brown & Maydeu-Olivares, 2011) within structural equation modeling (SEM) and the Markov chain Monte Carlo (MCMC) sampling method (Gilks et al., 1996). Although a full information maximum likelihood estimation is possible (Frick, 2023), the models are so complicated that the estimations are generally impractical. Additionally, existing models may not accommodate certain scoring assumptions or schemes for new FC item formats (an example will be provided later). This paper proposes a new approach, the ranking pattern approach, for building IRT models for FC items. This approach employs the adjacent category logit function (van der Ark, 2001), which is used in the generalized partial credit model (GPCM; Muraki, 1993). We propose a new model under this approach for the dominance process, which we refer to as the multidimensional generalized partial preference model (MGPPM). The model under the new approach is more flexible regarding FC scoring schemes and assumptions, more statistically elegant, and more efficient for model estimations.

The remainder of the paper is organized as follows. First, we provide a brief review of the two existing modeling approaches, followed by the research statements. Second, we present the ranking pattern approach and the MGPPM model, along with their relationship to existing models. Third, we describe a maximum marginal likelihood estimation with an expectation-maximization algorithm (MML-EM; Bock & Aitkin, 1981; Fu, 2019) and a Bayesian estimation with the MCMC sampling method for the MGPPM. Fourth, we conduct a simulation study to assess parameter recovery from MML-EM for triplets and tetrads (FC items with three and four statements, respectively), as well as from MCMC for triplets. We also compare MGPPM’s MML-EM estimations on simulated triplet data to Triplet-2PLM’s and TIRT model’s estimations. Fifth, we apply the MGPPM to one real dataset with triplets and compare its MML-EM estimation to those based on the Triplet-2PLM and TIRT model. Finally, we summarize the findings and discuss their implications, limitations, and future research directions.

## 2 Existing IRT models for FC items

### 2.1 Sequential selection

In a sequential selection process, test takers select the first favorable statement from the 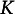 (>1) statements in an item, the second favorable from the remaining 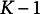 statements, and so on, until all statements are ranked. A probability model models this process. For example, in a triple item ( 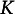 = 3) containing three statements (Statements 1–3), the ranking pattern ( 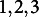 ) refers to Statement 1 as the most favorable choice and Statement 3 as the least favorable. Denote 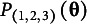 as the probability of the ranking pattern ( 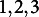 ) conditional on the column vector of the latent trait score(s) 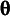 that the item measures. Then, based on the assumption of the sequential selection,
(1)

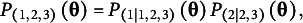

where 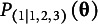 is the conditional probability of selecting Statement 1 from the three statements and 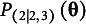 is the conditional probability of selecting Statement 2 from the remaining Statements 2 and 3, both given that only one statement is selected from the available three or two statements.

Denote the selection of a statement as 1 and 0 otherwise. If multiple selections are allowed, rather than forcing the choice of one selection, eight selection patterns would exist on the three statements in the first step. However, only one selection is allowed in each step of the sequential selection. Thus, only three selection patterns, (1,0,0), (0,1,0), and (0,0,1), are legitimate. Let 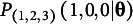 be the conditional joint probability of three decisions made in the first step: selecting Statement 1 and declining Statements 2 and 3. Likewise, define 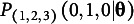 and 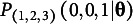 as the conditional joint probabilities of selecting Statements 2 and 3, respectively, in the first step while declining the others. Obviously, 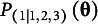 is proportional to 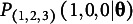 . Assuming the ratios among the three joint probabilities do not change after test takers are limited to the three selection patterns, we can normalize 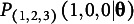 and denote it as 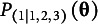 ,
(2)

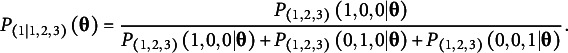



Similarly,
(3)

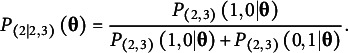



Similarly, for a pair item containing Statements 1 and 2, it is a one-step selection, and the normalized probability of selecting the first statement is
(4)

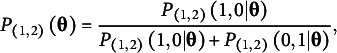

which is Andrich’s basic model for FC items (Andrich, 1995).

By assuming independent judgment of individual statements, the joint distributions can be simplified as the product of the probabilities of selecting or not selecting individual statements. For example, 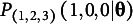 is simplified as
(5)

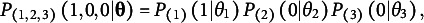

where 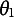 , 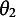 , and 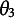 refer to the latent trait scores measured by Statements 1–3, respectively. The probability of selecting/not selecting a statement, 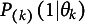 or 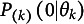 , is a function of the statement’s utility 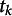 ( 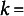 1, 2, or 3), and 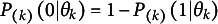 . Two popular processes are assumed to underlie the selection of individual statements: the dominance and ideal-point processes. The dominance process assumes a monotonic relationship between the degree of agreement on a statement and the underlying trait it measures. In contrast, the ideal-point process assumes that a statement has an optimal point on the trait continuum, which most likely endorses the statement, and the endorsement likelihood decreases as the trait level departs from this optimal point above or below. Different probability models have been proposed to model the two processes of judging statements. The notable model for the ideal-point process is the generalized graded unfolding model (GGUM; Roberts et al., 2000), whereas that for the dominance process is the two-parameter logistic IRT model (2PLM; Birnbaum, 1968). The response function of the 2PLM is
(6)

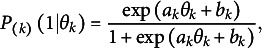

where 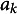 is the statement 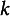 ’s slope/discrimination parameter and 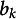 is the intercept. In Equation 6, the statement’s utility is represented by
(7)

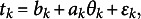

where the errors 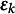 follow an independent and identical logistic distribution with a mean of 0 and a common variance across statements. Equation 6 is the cumulative distribution function of 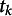 .

The IRT models under the sequential selection process were collectively named “Rank models” (de la Torre et al., 2012; Fu et al., 2025). The specific models under the Rank models can be distinguished by the block size (i.e., the number of statements in an item) and the probability function of selecting individual statements. Notable models for the ideal-point process include Stark et al.’s (2005) multi-unidimensional pairwise preference (MUPP) model, combined with the dichotomous version of GGUM (MUPP-GGUM) for pairs and the Triplet-GGUM for triplets (Joo et al., 2018; Lee et al., 2019). For the dominance process, the MUPP-2PLM (Morillo et al., 2016) for pairs is a special case of the commonly known compensatory multidimensional two-parameter logistic IRT model (M2PLM; von Davier, 2008), and its response function is
(8)

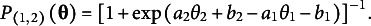



The response function of the Triplet-2PLM (Fu et al., 2025; Zheng et al., 2024) for triplets is
(9)

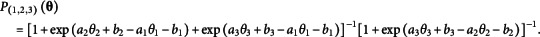



Equations 8 and 9 can be derived based on Equations 1–6. Note that in Equations 8 and 9, only one and two, respectively, of the intercepts ( 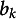 ) can be identified. Brown (2016) has pointed out that the Rank models with logistic response functions for individual statements are consistent with Luce’s choice axioms (Luce, 1959) and the Bradley–Terry model (Bradley, 1953; McFadden, 1973).

The estimations of the Rank models have been developed under the full-information approach. The MUPP-2PLM is a special case of traditional multidimensional IRT models; thus, the MML-EM and MCMC estimation methods developed for traditional multidimensional IRT models can be applied directly to estimate the MUPP-2PLM. Fu et al. (2025) and Zheng et al. (2024) developed the MML-EM method for the Triplet-2PLM. Lee et al. (2019) estimated the MUPP-GGUM and Triplet-GGUM using MCMC, and Fu (2025) calibrated the MUPP-GGUM using the MML-EM method.

### 2.2 Thurstone’s law of pairwise comparison

According to Thurstone’s (1927) law of comparative judgment, for a pair of statements, statement selection is determined by the difference between the two statements’ utilities:
(10)

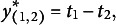

where 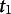 and 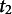 are independent conditional on latent traits that the two statements measure, based on the assumption of independent judgments of individual statements. If 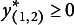 , Statement 1 is selected; otherwise, Statement 2 is selected. Statement utilities can represent an ideal-point or dominance process. Zhang, Tu, et al.’s (2024) generalized that Thurstonian unfolding model (GTUM) is an example of an ideal-point model under Thurstone’s approach. The most popular model, the Thurstonian IRT (TIRT) model (Brown & Maydeu-Olivares, 2011), is a dominance model. In the TIRT model, each statement’s utility is represented by a one-factor model,
(11)

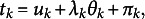

where 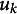 is the intercept, 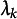 is the factor loading, 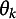 is the latent trait score that the statement measures, and 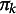 is the independent and identically normally distributed error with a mean of 0 and a variance of 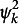 . Note that Equation 11 has the same basic form as Equation 7; however, we use different notations to facilitate comparison between the models under the two different modeling approaches. The difference between the two statement utilities is
(12)

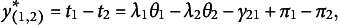

where 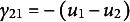 is the threshold. The probability of selecting Statement 1 is the probability of 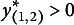 and modeled by a normal ogive function 
(13)

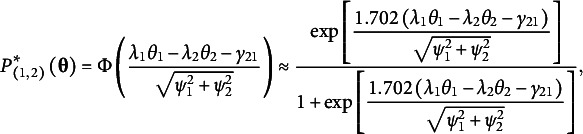

where 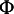 is the standard normal cumulative function. For pairs, the model can be identified by setting the error variance of 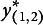 to a constant, for example, 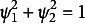 . The first part of Equation 13 is the TIRT model’s item response function, a special case of the compensatory two-parameter normal ogive multidimensional IRT model (McDonald, 1997). The approximation between the normal ogive function and the logistic function in Equation 13 is well known (Baker & Kim, 2004). The second part of Equation 13 is equivalent to the MUPP-2PLM’s item response function (Equation 8) by parameter transformations. Furthermore, if we assume 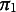 and 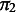 follow a Weibull distribution so that 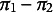 follow a logistic distribution, the probability of 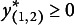 can be directly modeled by the MUPP-2PLM.

For items with more than two statements (i.e., 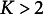 ), the TIRT model recodes a ranking pattern into 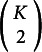 ranking patterns of pairs. For a triplet, the response pattern of the three statements (Statements 1–3) can be separated into three statement pairs: Pair 1 (1, 2), Pair 2 (1, 3), and Pair 3 (2, 3). The response functions (marginal probabilities of ranking patterns) for the three pairs are 
(14)

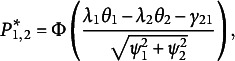



(15)

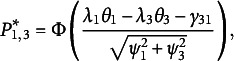



(16)

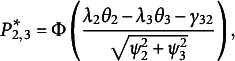

where 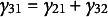 .^1^ Equations 14–16 can be identified by fixing the error variance of one statement, for example, setting the error variance of the first statement ( 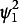 ) to 1. The TIRT model considers the local dependence among the three pairs stemming from a triplet by estimating the covariances among the three pairs’ 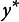 s: 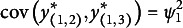 , 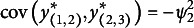 , and 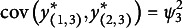 . The TIRT model is set up with mean and covariance structures under structural equation modeling (SEM) and estimated using a limited information method, unweighted least squares with mean and variance-corrected Satorra–Bentler goodness-of-fit tests (ULSMV). However, local dependence is considered only in item parameter estimation; for estimating latent trait scores, local independence is currently assumed in Equations 14–16.

The MUPP-2PLM model assumes local independence of item responses to pairs and is estimated using a full-information method. Thus, this model is unsuitable for estimating an item with more than two statements when it is separated into pairs. Some researchers have proposed adding additional random parameters to the logistic item response functions to account for dependence among pairs, as seen in Bürkner et al. (2019, Equation 4) and Zhang, Tu, et al. (2024, Equation 14). However, the additions make the models more complicated and harder to estimate (especially for maximum likelihood estimation).

For items with more than two statements ( 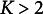 ), the difference in the modeling approaches between the Rank and Thurstonian models becomes apparent. For the Thurstonian models, the probability of a ranking pattern ( 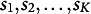 ), where 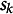 denotes the statement ID with rank 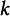 , is the joint distribution of selecting the first statements in the 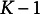 pairs, ( 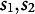 ), ( 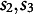 ), …, ( 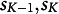 ). For example, for a triplet, the probability of a ranking pattern (1, 2, 3) under the TIRT model is the joint probability of selecting the first statements in the two pairs, (1,2) and (2,3), and given by (Maydeu-Olivares, 1999) 
(17)

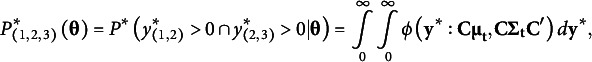

where 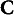 is the 2-by-3 contrast matrix 
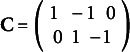
 ; 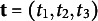 ; 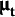 and 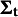 are the mean vector and covariance matrix of 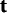 ; 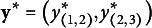 follows a bivariate normal distribution with a mean vector 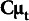 and covariance matrix 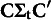 ; 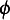 is the bivariate normal density function. It is obvious that Equation 17 and Equation 1 (the Rank models’ item response function for a triplet) are different: For the Rank models, Equation 1 can be rewritten using 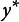 s as
(18)

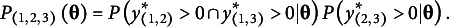



Equation 17 represents a full joint distribution of a ranking pattern (1, 2, 3), while Equation 18 simplifies this joint distribution by separating it into two independent distributions. Specifically, in Equation 18, the comparison between Statements 2 and 3 is independent of Statement 1, which indicates that the Rank models hold a stronger assumption and, thus, are simpler than the Thurstonian models. Compared Equation 9 to Equations 14–16, the TIRT model for a triplet has two more parameters (i.e., error variances of two statements) than the Triplet-2PLM.

The FC item format discussed so far is dichotomous; test takers must select one statement from two or more statements. Sometimes, this format may be challenging because a test taker may like two statements equally. In addition, this format does not capture the degree to which a test taker prefers one statement over another. In contrast, Likert scales use graded responses (e.g., “strongly disagree,” “disagree,” “neutral,” “agree,” “disagree”) to gain more information on a statement and, thus, may generate more reliable trait scores than the dichotomous FC item format (Bürkner, 2022). To address this issue, Brown and Maydeu-Olivares (2018) extended the TIRT model to accommodate the graded FC item format (e.g., “I strongly like Statement 1,” “I like Statement 1,” “I like both statements equally,” “I like Statement 2,” “I strongly like Statement 2”). The graded TIRT model follows the approach used by the graded response model (GRM; Samejima, 1969) and employs 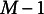 thresholds, 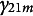 ( 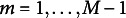 ), to divide the continuous difference of item utilities, 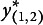 , into 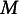 ordinal response categories. Denote a test taker’s response as 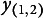 . Then, the item response function of 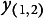 is,
(19)

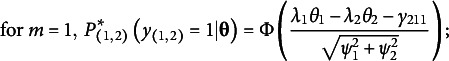



(20)

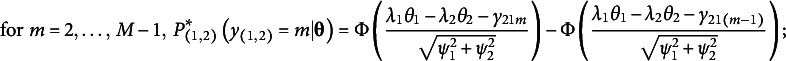



(21)

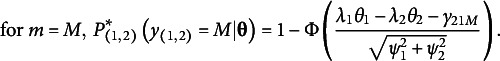



Zhang, Tu, et al. (2024) used the same approach to handle the graded FC item format in their GTUM. Although theoretically, the graded Thurstonian models for FC items can calibrate items with any block sizes, the graded response format seems only practically feasible for pair items. Brown and Maydeu-Olivares (2018) and Zhang, Luo, et al. (2023) reported improved reliabilities for using the graded format in FC items compared to the dichotomous format.

## 3 Purpose of the current study

We propose a new IRT approach to calibrate FC items’ responses differently from those presented previously. In the new approach, we discard the assumption of independent judgments on individual statements and instead work directly on a ranking pattern of an item. We refer to it as the ranking pattern approach. Focusing on the dominance process, we propose the multidimensional generalized partial preference model (MGPPM) inspired by the scoring function in the GPCM. As detailed later, the MGPPM is a well-defined model with advantages over the Rank-2PLM and the TIRT model in terms of estimation efficiency and model flexibility.

Ideal-point models are useful for measuring social and political attitudes, where intermediate statements are often meaningful. However, dominance models are generally simpler and easier to estimate than ideal-point models (Fu et al., in press). In personality assessments (especially with normal populations), dominance models work generally well and are the most commonly used models.

## 4 Model presentation

In the ranking pattern approach, we work on ranking patterns directly rather than decomposing the probability of a ranking pattern into the response functions of individual statements. We assume that each statement 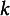 at each rank 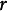 (= 1,…, 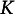 ; 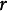 = 1 refers to the most favorable statement) in an item with 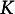 (>1) statements has a utility 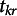 , and each rank 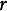 is assigned a score of 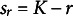 . For the last rank 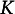 (i.e., the least favorable statement), we set 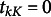 , and for the second-to-last rank 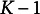 , we define
(22)

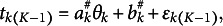

where 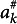 is the slope/discrimination parameter associated with statement 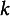 , 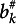 is the intercept, and 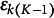 is the independent and identically distributed (IID) error with a mean of 0. For the other rankings ( 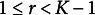 ), there are options to define their utilities.

The primary option assumes that the advancement of one rank increases the statement’s utility by 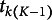 , and thus,
(23)

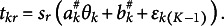



Equation 23 is analogous to the use of the scoring function in the GPCM. Then, the utility of a ranking pattern with ID 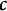 is the sum of the utilities of all ranked statements,
(24)

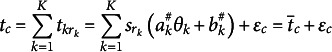

where 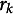 refers to the statement 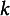 ’s rank; 
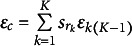
 is the IID error with a mean of 0 for ranking pattern 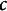 ; and 
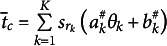
 is the mean of 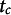 . For an FC item with 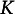 statements, there are 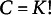 ranking patterns. Assume 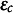 follows a Weibull distribution, and then, the probability of ranking pattern 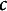 can be represented by
(25)

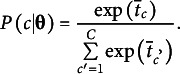



Among 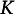 intercepts, 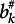 s, in Equation 25, one of them cannot be identified. It is convenient to set Statement 1’s intercept 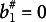 . If all 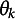 s refer to a common trait, then one of the 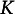 slopes, 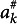 , cannot be identified either and needs to be fixed. Note that Equation 25 has the same form as McFadden’s (1973) extended Bradley–Terry model (see Brown, 2016, Equation 16); the difference is that the Bradley–Terry model works on the utility of each statement, while ours builds on the utility of each ranking pattern.

Let us apply the model to triplets as a concrete example. For a triplet, there are six ranking patterns, as shown in Table 1, and they are coded as 1–6 (i.e., ranking pattern ID 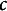 ) in the observed data. For example, Score 1 is associated with the ranking pattern (1,2,3): Statement 1 is ranked first, Statement 2 is ranked second, and Statement 3 is ranked last.

**Table 1:** Possible ranking patterns for an item with three statements: 1, 2, and 3

Coded score	1	2	3	4	5	6
Ranking pattern	1,2,3	1,3,2	2,1,3	2,3,1	3,1,2	3,2,1

The mean utilities of the six ranking patterns are represented by the following: 
(26)

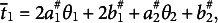



(27)

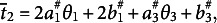



(28)

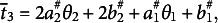



(29)

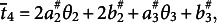



(30)

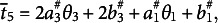



(31)

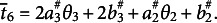

Each ranking pattern’s mean utility is the sum of the utilities of the three statements at their respective rankings. For example, Ranking Pattern 1’s mean utility is the sum of the utilities of the first statement at rank 1 ( 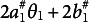 ), the second statement at rank 2 ( 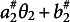 ), and the third statement at rank 3 (0). Then, the probability of each ranking pattern is defined by Equation 25. Note the logarithm of the ratio of the probabilities of two ranking patterns (i.e., the conditional logit function given two ranking patterns), for example,
(32)

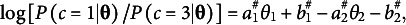



(33)

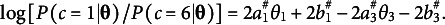



In Equation 32, the increase in Statement 1’s rank from 2 to 1 is accounted by 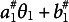 , and the decrease in Statement 2’s rank from 1 to 2 is accounted by 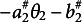 . In Equation 33, the rank changes for Statements 1 and 3 are two rank levels and are thus represented by 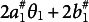 and 
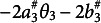
 , respectively. Equations 32 and 33 are analogous to the conditional logit functions in GPCM and reflect a partial credit for each increment of one rank level. Therefore, we refer to it as the multidimensional generalized partial preference model (MGPPM). Among the three intercepts, 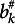 s, in Equations 26–31, only two of them can be estimated, and we may set 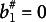 to identify the model. If all statements measure the same trait, then one of the three slopes, 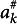 s, also needs to be fixed.

**Figure 1:**
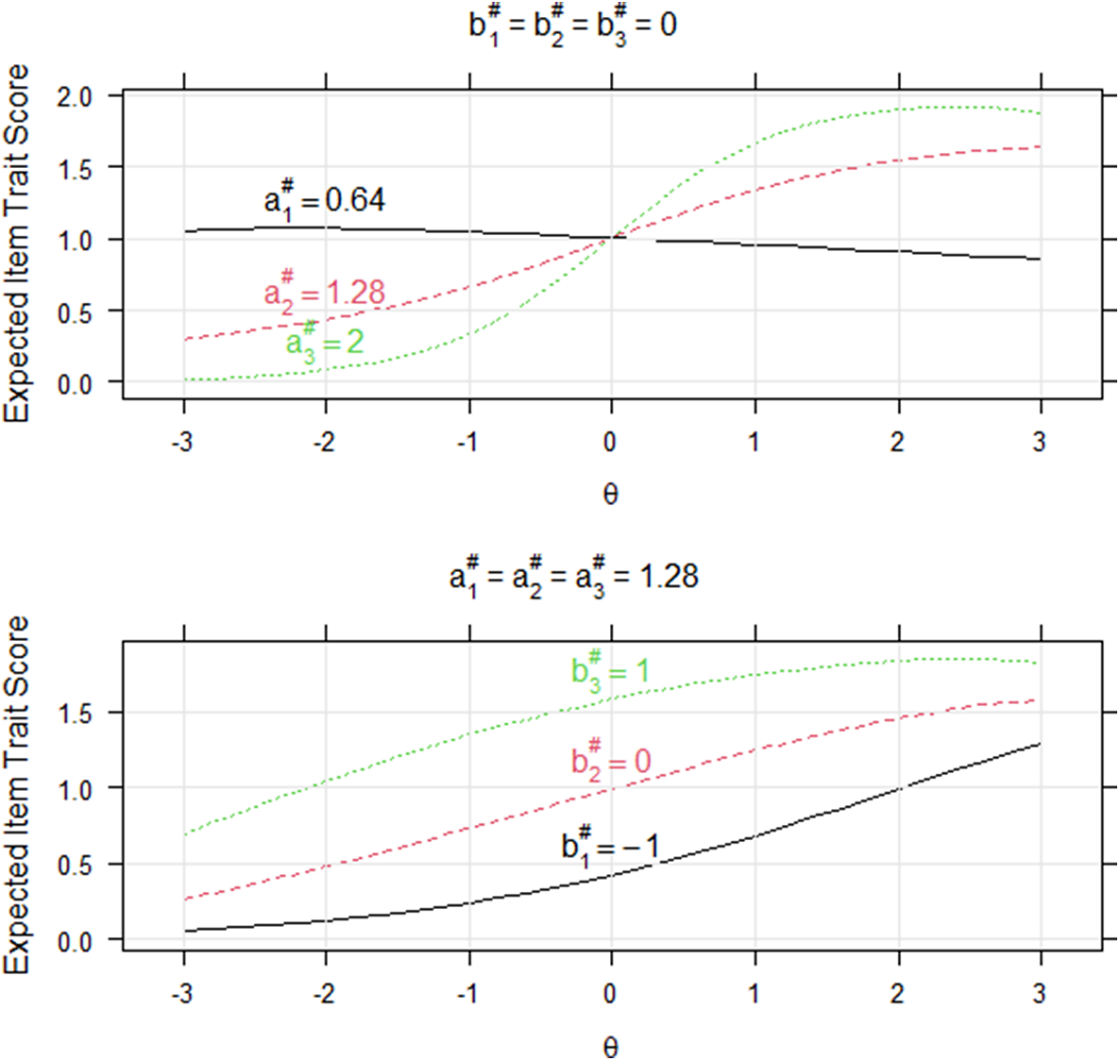
Item trait characteristic curves of two triplet examples.

The interpretations of 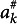 and 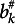 are like those of the slope and intercept parameters in the 2PLM/GPCM. The slope and intercept parameters control the steepness and location, respectively, of the item trait characteristic curve (ITCC; Fu, Tan, & Kyllonen, 2024b). Figure 1 compares the three ITCCs in two triplets. An ITCC plots expected item trait scores against the latent trait scores. For a ranking pattern of a triplet, we assign a score of 2 to the trait measured by the first rank statement, 1 for the second rank, and 0 for the last rank. The expectation is based on the joint distribution of the three traits in a triplet. For example, let 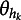 denote a score point 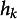 of the trait measured by Statement 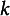 . Then, the expected item trait score at a score point 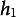 of the trait measured by Statement 1 in an item ( 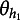 ) is computed as
(34)

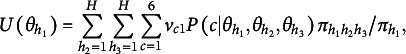

where 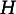 (= 31) is the number of equally spaced points from −3 to 3 for each trait; 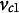 (= 0, 1, or 2) is the trait score measured by Statement 1 associated with ranking pattern 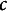 (Table 1); 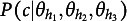 is the probability of ranking pattern 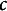 conditional on a trait score vector ( 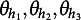 ) (Equation 25); 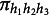 is the weight of a trait score vector ( 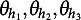 ) that is the normalized density from the multivariate normal distribution; and 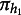 is the weight of 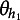 that is the normalized density of the normal distribution. The triplet in the upper panel of Figure 1 contains three statements with increasing slopes of 0.64, 1.28, and 2 and a common intercept of 0. One can see that the ITCCs become steeper as the slopes increase. In contrast, the triplet in the lower panel of Figure 1 contains three statements with a constant slope of 1.28 and increasing intercepts of −1, 0, and 1. In this case, the three ITCCs have similar shapes but different locations, following the same order as the intercepts. A statement with a higher intercept is more likely to result in a higher rank.

The primary option for the utility of a ranked statement assumes that the utility increase is a constant for each advancement of one rank on the statement for a test taker. An alternative option will allow the utility increase to vary for each rank increment. For this option, we define the utility of a statement with rank 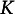 as 0 and with rank 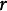 ( 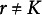 ) as
(35)

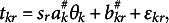

where 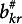 is the intercept associated with statement 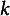 with rank 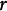 and 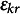 is the IID error with a mean of 0. In Equation 35, the utility increase for a test taker with a one-level increase from a rank level differs from that with a one-level increase from another because the utility at each rank level is associated with a unique intercept 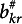 . For this alternative option, two of the 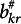 s are not identified and must be fixed. If all statements measure a common trait, two of the 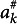 s are also not identified and need to be fixed. This alternative model is more complicated and has 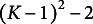 more parameters than the primary model for 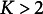 . Since the primary model is more parsimonious and practical, we focus on it in this article.

### 4.1 Applications to other item formats

Besides the rank item format (RANK), the MGPPM can be easily applied to other item formats or scoring schemes. Below, we discuss the applications of the MGPPM to four alternative formats: PICK (i.e., pick the statement that is most descriptive of a test taker), MOLE (i.e., choose the statements that are the most and least descriptive of a test taker) (de la Torre et al., 2012), graded response, and a newly proposed hybrid forced-choice/Likert (HFCL; Williams et al., 2025).

#### 4.1.1 PICK and MOLE

The PICK and MOLE formats are alternatives to the RANK format, designed to reduce test takers’ cognitive burden for FC items with larger block sizes. See Hontangas et al. (2015) for examples of these two formats in real questionnaires. The response data from the PICK and MOLE formats can be considered equivalent to that from the RANK format with planned missing data. Thus, for an IRT model, the item response functions for the PICK and MOLE formats can be derived from that for the RANK format by accounting for planned missing data (Yousfi, 2025). That is, the response function of an item response 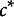 from the PICK or MOLE format can be written as
(36)

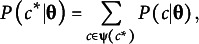

where 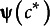 is the set of full responses from the RANK format that are not in contradiction with item response 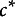 from the PICK or MOLE format. For example, for a tetrad with Statements 1–4, the probability of choosing Statement 1 from the PICK format is the sum of the probabilities of the six ranking patterns with Statement 1 as rank 1 from the RANK format, and the probability of selecting Statements 1 and 2 as the most and least descriptive, respectively, from the MOLE format is the sum of the probabilities of the four ranking patterns with Statements 1 and 2 as ranks 1 and 4, respectively, from the RANK format.

#### 4.1.2 Graded response

The MGPPM shows great flexibility in handling the graded FC item format. For example, for a pair item with five graded response categories, we can define the scoring function for both statements as shown in Table 2. Then, by treating each response category as a ranking pattern, the MGPPM for a graded pair item is defined by Equations 23–25. If we use the primary option (Equation 23), this model has the same number of model parameters as the MUPP-2PLM. If we use the alternative option (Equation 35), this model has the same number of model parameters as the graded TIRT model in Brown and Maydeu-Olivares (2018), and their relationship is similar to that between the traditional GPCM and GRM.

**Table 2:** Scoring function for a pair item with five graded response categories

			I like both		
	I strongly like	I like	statements	I like	I strongly like
Response	Statement 1	Statement 1	equally	Statement 2	Statement 2
Statement 1 score ( 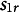 )	2	1	0	0	0
Statement 2 score ( 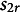 )	0	0	0	1	2

#### 4.1.3 Hybrid Forced-Choice/Likert (HFCL)

Williams et al. (2025) proposed a new FC item format, a hybrid of the FC and Likert item formats (HFCL). An HFCL item includes a block of two or more statements, similar to an FC item. However, unlike an FC item, an HFCL item asks test takers to rate each statement separately using multilevel Likert responses (e.g., scores 1 to 4 representing strongly disagree, disagree, agree, and strongly agree), with the condition that no rating is allowed to be equal between two statements. This hybrid format aims to (a) reduce test takers’ cognitive burden on answering traditional FC items and enhance the test-taking experience, (b) collect more response information by the graded response format, and (c) maintain the fake resistance achieved by traditional FC items to some extent by not allowing equal rating among statements. Williams et al. (2025) reported a more positive user experience with the HFCL format than with traditional FC formats in aspects such as accuracy, fairness, ease of use, and enjoyment, as indicated by respondent feedback data. Figueiras et al. (2025) conducted a comparison study on faking resistance between the HFCL and Likert formats, finding that the HFCL was generally more resistant to faking than the Likert.

Research is underway to find the best IRT scoring model for HFCL items. The MGPPM appears to be the most promising option due to its flexibility and preservation of all rating data. To calibrate an HFCL item by the MGPPM, we can set each statement’s scoring function ( 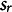 ) to be its rating score minus 1. Then, either the primary model (which has the same number of model parameters as the Rank-2PLM) or the alternative model applies. Brown and Maydeu-Olivares’s (2018) graded TIRT model can also calibrate an HFCL item by separating the item (if the block size is larger than two) into pairs and treating the rating score differences between the two statements as graded responses. However, the graded TIRT model discards individual statements’ rating information when transforming rating scores into score differences. For example, the rating scores, 1 and 2, of two statements are treated the same as 3 and 4 in the graded TIRT model, which may not be desirable in some cases. In contrast, the MGPPM considers the rating score of each statement; thus, each rating score pattern in a hybrid item has a unique effect.

### 4.2 Relation to Thurstone’s models and rank models

The basic analysis unit of the ranking pattern approach is the utility of a statement at a rank level. Thus, the utility is within the context of comparing with other statements. In contrast, the basic analysis unit of the Rank and Thurstone’s models is the utility of a statement that is assumed to be independent of other statements. The utility of a statement is arguably constant no matter which test format (e.g., Likert vs. forced choice) or context (e.g., a statement in different FC items) this statement is administered with. This difference has significant implications for test assemblies, which we will explore further in the discussion section.

For all models, the probability of a ranking pattern is based on the joint distribution of the ranking pattern. However, the Rank models and primary MGPPM make assumptions to simplify the joint distribution. The Rank models decompose the joint probability into the product of 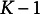 independent probabilities, where statements are ranked sequentially. The primary MGPPM assumes that the differences in the probabilities of a statement’s two consecutive rankings are constant for a test taker, while the alternative MGPPM does not make this assumption. However, these assumptions only affect items with more than two statements. For pair items, the item response functions are the same or equivalent for all models, given that their formulations of statements’ utilities are the same or equivalent. For items with block sizes larger than two, the Rank-2PLM and the primary MGPPM have the same number of model parameters, while the TIRT model and the alternative MGPPM have the same number of model parameters and are more complex than the previous two models.

All three approaches provide flexible formulations to accommodate block sizes and cognitive processes underlying statement judgments (i.e., ideal point and dominance). Although not discussed here, the ranking pattern approach can handle the ideal-point process by using an ideal-point formulation of the utility of a statement at a rank. By modeling the utility at the ranking pattern level, the ranking pattern approach is more flexible in incorporating different assumptions into models than the other two approaches. Although we only discuss the two options for representing a ranked statement’s utility, many other options are possible. Using the ranking pattern approach, researchers can easily test different assumptions about a ranking process by adjusting the scoring function of a statement. For instance, one may assume that the advancement from rank 2 to 1 represents twice the utility increase from rank 3 to 2 (rather than once, as in the primary MGPPM). Additionally, the ranking pattern approach can be applied directly to the graded response format and rating score pattern, as previously demonstrated. Although Thurstone’s approach can estimate the graded response format, it cannot handle rating score patterns, whereas the sequential selection approach cannot handle both due to its modeling assumption.

The TIRT model is traditionally estimated using a limited information method under SEM, while the Rank models and MGPPM are estimated using a full information method, such as the maximum marginal likelihood estimation (MMLE). The TIRT can be estimated using the MMLE with Equation 17 as the response function. Frick (2023) employed Equation 17 and maximum likelihood estimation to estimate person parameters with known item parameters, thereby avoiding the local independence assumption in the person parameter estimation of the TIRT model. However, we have not found a study on the MMLE of the TIRT model’s item parameters. Under some conditions, a limited information method may be inferior to a full information method (Forero & Maydeu-Olivares, 2009). Nevertheless, all models can be easily set up for Bayesian estimations using the MCMC sampling method. The MGPPM is more elegant than the TIRT and Rank-2PLM as its conditional logit functions (e.g., Equations 32 and 33) are more concise and straightforward.

Like the traditional IRT models for cognitive tests, the models under the ranking pattern approach are statistical models to estimate the probabilities of item responses, rather than modeling a cognitive decision process or strategy that test takers are assumed to use to rank the statements in an item, because it is too cognitively demanding for test takers to consider all possible ranking patterns for FC items with a block size larger than two. Test takers likely use a heuristic strategy to simplify the ranking process. For triplets, Sass et al. (2020) showed evidence that test takers used pairwise comparisons to rank statements in a think-aloud task. However, this study also reported that 24% of the participants had difficulty remembering the information of the three statements in a triplet when making pairwise comparisons. It is conceivable that more test takers may find the pairwise comparison approach cognitively challenging when ranking four or more statements. In this respect, the sequential selection approach is more likely to be a strategy most test takers apply when responding to FC items with large block sizes. Further research is needed on the cognitive decision processes underlying FC items.

## 5 Model estimation

### 5.1 MML-EM estimation

Suppose in an FCQ, a total of 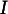 test takers respond to 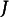 items. Let 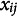 denotes test taker 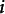 ’s nominal score (i.e., ranking pattern ID 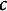 ) on item 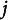 (Table 1); 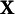 is the 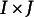 score matrix representing the total observed data. 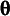 represents a random sample of latent trait score vectors from the test-taker population with a standard multivariate normal distribution with a 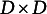 correlation matrix 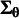 . Fixing the population means and standard deviations of 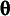 is a way to identify the model. 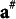 and 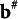 are the column vectors of slopes and intercepts, respectively. 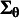 is the intertrait correlation matrix. Assume that 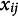 is locally independent conditional on item parameters and 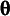 . Then, the observed data likelihood is a marginal likelihood
(37)

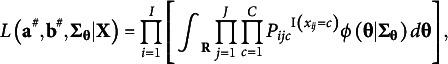

where 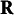 is the D-dimensional area of integration, 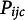 is the probability of a coded score (ranking pattern) 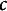 ( 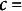 1,…, 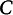 ) (Equation 25), 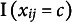 is the indicator function with a value of 1 if 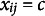 and 0 otherwise, and 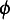 is the standard multivariate normal density function with a 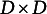 correlation matrix 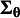 . Because directly maximizing the observed likelihood is difficult, a common solution is the EM algorithm (Bock & Aitkin, 1981) that works on the complete data log likelihood instead,
(38)

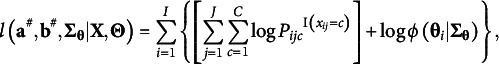

where 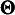 is the 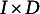 latent trait score matrix for all test takers, 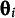 is test taker 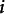 ’s latent trait score vector including 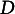 traits, and 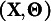 are the complete data with observed data 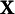 and missing data 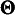 . The EM algorithm involves an iterative process that repeatedly executes two steps: an E step and an M step.

In the E step, the expectation of the complete data log likelihood with respect to the posterior distribution of missing data (i.e., trait scores) is estimated and serves as the EM map. The expectation process involves approximating the posterior distribution of trait scores. For a small number of traits (e.g., fewer than four), the posterior distribution can be approximated by quadrature points, such as the Gauss–Hermite quadrature points (Davis & Rabinowitz, 1984), the adaptive variants (Schilling & Bock, 2005), or the equidistant points within a range (e.g., −6 to 6). However, a typical FCQ includes more than three traits. The quadrature point method is not practical for high-dimensional traits due to the enormous computing memory it demands. The alternative is the stochastic approach, for example, the Metropolis-Hastings Robbins-Monro (MHRM) algorithm (Cai, 2010), which uses MCMC to draw random samples from the posterior distribution of trait scores.

In the M step, the EM map is maximized with respect to item parameters and distributional parameters of traits using the Newton–Raphson (NR) method (Atkinson, 1989) or a quasi-Newton method, for example, the BFGS algorithm (Broyden, 1970). The MHRM algorithm employs a method similar to the NR method to search for model parameters that maximize the EM map using the drawn samples at the E step.

The MML-EM method is a standard estimation method widely applied to IRT and many other models. The R package *mirt* (Chalmers, 2012) implemented a general-purpose MML-EM method for IRT models, which can be used to estimate existing IRT models and users’ newly proposed IRT models. For conciseness, we do not go into the details of the MML-EM method here. Interested readers are referred to Fu et al. (2025), which provides the technical details of the MML-EM method for estimating the Triplet-2PLM.

### 5.2 MCMC estimation

The MCMC is a sampling-based Bayesian estimation method that draws model parameters from their posterior distribution(s). The joint posterior distribution of all model parameters conditional on item responses in the MGPPM under the Bayesian framework is the product of the prior distributions of all parameters and the likelihood function,
(39)

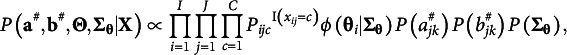

where 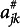 is the slope of statement 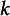 in item 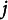 ; 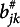 is the intercept of statement 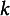 in item 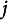 ; 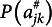 , 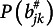 , and 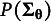 are the prior distributions of the slope, intercept, and intertrait correlations, respectively. A sampler, such as the No-U-Turn sampler (Hoffman & Gelman, 2014) implemented in the R package *rstan* (Stan Development Team, 2024), can be used to draw samples from the joint posterior distribution (Equation 39).

## 6 Simulation study

### 6.1 Simulation design

We conducted a simulation study to inspect the parameter recovery of the MGPPM on triplets and tetrad. We also compared the MGPPM’s model fits and trait score estimations to those of the TIRT and Triplet-2PLM on the triplet data simulated based on the MGPPM. For each simulated dataset, we had 1000 test takers and five traits, with each trait measured by 12 statements. This resulted in 20 triplets and 15 tetrads. We used two repeated combinations of three out of five to define the trait measured by each statement in the 20 triplets; for the 15 tetrads, we used three repeated combinations of four out of five traits. Trait scores were drawn from a multivariate normal distribution with a mean vector of 0 and an intertrait correlation matrix that was an excerpt of five traits (Traits 1, 6, 8, 10, and 12) from the real data, which included 14 traits (see Table 3). The intercepts were drawn from a normal distribution with a mean of −0.05 and a standard deviation (SD) of 0.54. The distributional parameters were taken from our real data. The intercept of the first statement in each triplet is set to 0 for model identification.

**Table 3:** Intertrait correlation matrix from real data with 14 traits

Trait	1	2	3	4	5	6	7	8	9	10	11	12	13
2	0.54												
3	0.62	0.62											
4	0.29	0.33	0.31										
5	0.58	0.65	0.72	0.28									
6	0.41	0.34	0.36	0.16	0.51								
7	0.37	0.36	0.45	0.15	0.58	0.74							
8	0.52	0.21	0.21	0.01	0.23	0.43	0.22						
9	0.33	0.07	0.08	0.16	0.06	0.35	0.23	0.51					
10	0.41	0.27	0.38	0.29	0.33	0.34	0.48	0.24	0.49				
11	0.18	0.11	0.22	0.18	0.23	0.40	0.48	0.13	0.32	0.53			
12	0.26	0.13	0.18	−0.06	0.20	0.06	0.18	0.17	0.20	0.28	0.16		
13	0.43	0.25	0.38	−0.10	0.45	0.25	0.37	0.31	0.13	0.29	0.17	0.54	
14	0.46	0.19	0.27	−0.12	0.33	0.37	0.33	0.49	0.32	0.28	0.17	0.53	0.63

In this simulation study, we controlled two factors. The first one was the mean of the slopes. The slopes were drawn from a lognormal distribution with an SD of 0.29 and three levels of means on the normal scale: low (0.64), medium (1.28), and high (2). The SD and low mean were also taken from our real data. Previous studies (e.g., Bürkner et al., 2019; Morillo et al., 2016; Lee et al., 2019) have demonstrated that slopes significantly impact the estimations of IRT models for FC items, with high slopes associated with more accurate parameter estimates. The second one was the keyed direction of statements within an item. We considered two levels. In the “same” level, all statements within a triplet/tetrad had the same direction: the first ten triplets and eight tetrads had positive statements, and the second ten triplets and seven tetrads had negative statements by multiplying −1 with the generated slopes. In the “mixed” level, for triplets, one statement was changed to a different keyed direction from the other two in half of the 20 items in the same level. The change of keyed direction on statements was balanced in terms of the five traits and positive/negative items so that there were five positive and five negative items having the direction of one statement in each item changed, and for each trait, only one statement’s direction was changed within each of the five-item groups. For tetrads, the last two statements in 7 of the 15 items at the same level (items 1–3 and 12–15) were changed to a different keyed direction from the first two statements. This design of keyed direction changes also considered balancing statement directions in each trait. Previous studies (e.g., Brown & Maydeu-Olivares, 2011; Bürkner et al., 2019; Bürkner, 2022) have demonstrated that item direction is a crucial factor influencing item information, and including mixed-direction items in an FCQ enhances test reliability. In this design, we considered the proportion of mixed-direction items in a test at 0.5 and 7/15 levels for triplets and tetrads, respectively, which are probably too high in real testing. However, we intended to use a relatively high proportion level to facilitate checking the general impact of mixed-direction items on model estimations.

In sum, the simulation study has six crossed conditions (3 slope levels 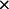 2 keyed directions) for each item format. We generated 35 replicate datasets in each condition. The item and person parameters are redrawn for each replicate dataset. Each triplet dataset was estimated using MML-EM and MCMC methods, while each tetrad dataset was estimated only using the MML-EM method. Each Triplet dataset was also estimated by TIRT with the ULSMV method and by Triplet-2PLM with the MML-EM method, and their model fits and trait score estimations were compared to those of MGPPM with the MML-EM method.

### 6.2 Estimation programs

The majority of the analyses was conducted in the R program (R Core Team, 2023). For the MML-EM estimations of MGPPM and Triplet-2PLM, we used the *mirt* package in R. Users can use the createItem function in *mirt* to define new item response functions. Then, the estimation and analysis modules in *mirt* can be used to estimate and further analyze the functions. The first and second derivatives of the MGPPM’s (Equations 25–31) and Triplet-2PLM’s (Equation 9) response functions for triplets were obtained using the symbolic derivative function, Deriv, in the R package *Deriv* (Clausen & Sokol, 2020). We used the MHRM algorithm implemented in *mirt* for the MML-EM estimations. For the MHRM algorithm, *mirt* estimates the MHRM standard errors (SE) of model parameter estimates; however, it carries out the SE estimation separately after the parameter estimation is done, and thus, it takes much longer to obtain SE estimates. To save running time, we modified the functions in *mirt* so that parameters and their SE estimations were performed simultaneously. The starting values of the discrimination parameters were set to 1 for positive statements and −1 for negative statements. All the starting values of the intercepts and intertrait correlations were set to 0 and 0.25, respectively. All trait score estimates were Maximum A Posterior (MAP) estimates. The other default settings in *mirt*, including the convergence criterion, were used for all estimations.

The TIRT model was estimated using the 64-bit *Mplus* 8.4 software (Muthén & Muthén, 1998–2017) under structural equation modeling with the ULSMV method and default settings. We fixed the error variance of the first statement ( 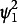 ) in a triplet to 1 to identify the model. We modified the functions in the R package *thurstonianIRT* (Bürkner, 2024) and used them to set up and run the TIRT models in *Mplus* from R. Once the item parameter estimates were obtained from *Mplus*, we set up an M2PLM in *mirt* and fixed the item parameters to be those estimates from *Mplus* (after parameter transformation). Then, the many functions in *mirt* could be used for further analyses. Note that in the estimation of the TIRT model in *Mplus*, the covariances among the three pairs stemming from a triplet were considered only in the item parameter estimation and not in the trait score estimation (i.e., the local independence assumed in Equations 14–16). Thus, the trait score estimates from the above setup of *mirt* are theoretically the same as those from *Mplus* because their item response functions and response data are the same. Frick (2023) and Maydeu-Olivares and Brown (2010) demonstrated that simplifying scoring had a minimal impact on the accuracy of latent score estimates.

The MCMC estimations of triplets were conducted using the *rstan* package (Stan Development Team, 2024). We applied weakly informative priors to the slopes and intercepts. For the slope prior, we used the lognormal distribution with a mean of 0 and an SD of 2 on the log scale. We assumed that the signs of slopes were known and fixed them during the estimations. For the intercept prior, we used a normal distribution with a mean of 0 and an SD of 3. For the intertrait correlation prior, we used the Lewandowski–Kurowicka–Joe (LKJ) distribution with a shape parameter of 4. Each estimation ran four chains, each with 8,000 iterations, with the first 4,000 iterations used as the burn-in period. For each parameter, the mean of the 16,000 final samples (4,000 samples per group) served as the estimate, and the SE of the estimate could also be estimated from the final samples. An estimation was deemed converged if the 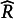 statistic (Gelman, 1996, p. 137) was smaller than 1.01, and the bulk effective sample size was larger than 400 for every estimated parameter.

The supplemental online materials (SOM) provide the R code to simulate triplet and tetrad item responses based on the MGPPM and estimate the models using the MML-EM method for both item formats and the MCMC method for triplets. In addition, the following R code is also provided in SOM: (a) estimating the Triplet-2PLM using the MML-EM method, (b) setting up and running the TIRT model in *Mplus* from R, and (c) setting up the TIRT model and estimating trait scores in *mirt* with fixed item parameters from *Mplus*’s estimates (after transformation).

### 6.3 Results: MGPPM’s parameter recovery

We checked the recovery of slopes, intercepts, intertrait correlations, and trait score estimates in the MGPPM. The following criteria were used: mean deviation (MD), mean absolute deviation (MAD), root mean square error (RMSE), mean relative deviation (MRD), and correlation (Cor) with true values.

#### 6.3.1 Triplets

Tables 4 and 5 show the parameter recovery results for the MML-EM and MCMC estimations of triplets, respectively. Only converged runs were included in the parameter recovery results. The converged rates were included in Tables 4 and 5. For the MML-EM method, only two runs in the same direction and one run in the mixed direction under the low-slope condition were not converged due to poor intertrait correlation estimates that caused the estimations to crash. There were more nonconvergent runs in the MCMC estimations (probably due to the different convergence rule from the MML-EM method): four runs in the same direction in the low- and high-slope conditions, nine runs in the same-direction-medium-slope condition, and two runs in the mixed-direction-high-slope condition. For the MCMC runs, the same-direction runs were more difficult to converge than the mixed-direction runs.

**Table 4:** Triplet MML-EM: Parameter recovery results

	Keyed direction				Intertrait						Mean of
Slope	(convergence rate)	Criterion	Slope	Intercept	correlation	Trait 1	Trait 2	Trait 3	Trait 4	Trait 5	Traits 1–5
Low	Same (94%)	MD	0.00	0.00	0.11	−0.01	−0.01	−0.01	0.00	0.01	−0.01
				MAD	0.09	0.05	0.11	0.52	0.53	0.53	0.53	0.52	0.53
				RMSE	0.11	0.06	0.12	0.66	0.66	0.67	0.66	0.66	0.66
				MRD	0.00	−0.05	0.57	−0.05	−0.06	−0.04	−0.06	0.07	−0.03
				Cor	0.99	0.99	0.95	0.75	0.75	0.74	0.75	0.75	0.75
		Mixed (97%)	MD	0.00	0.00	0.02	−0.01	−0.02	0.00	−0.01	0.01	−0.01
				MAD	0.06	0.05	0.04	0.42	0.43	0.44	0.43	0.44	0.43
				RMSE	0.08	0.06	0.05	0.53	0.55	0.55	0.54	0.56	0.55
				MRD	−0.01	0.00	0.08	−0.04	−0.02	0.02	−0.04	0.01	−0.01
				Cor	0.99	0.99	0.96	0.85	0.84	0.84	0.84	0.83	0.84
Medium	Same (100%)	MD	−0.01	0.00	−0.08	0.00	0.00	0.00	−0.01	0.01	0.00
				MAD	0.10	0.06	0.08	0.48	0.47	0.48	0.47	0.47	0.48
				RMSE	0.12	0.08	0.09	0.61	0.59	0.60	0.60	0.60	0.60
				MRD	0.00	0.01	−0.38	0.03	−0.02	0.01	−0.03	0.04	0.01
				Cor	1.00	0.99	0.97	0.79	0.81	0.80	0.80	0.80	0.80
		Mixed (100%)	MD	0.00	0.00	0.01	0.00	−0.01	0.00	−0.02	0.00	0.00
				MAD	0.08	0.07	0.03	0.31	0.31	0.31	0.31	0.32	0.31
				RMSE	0.10	0.09	0.03	0.40	0.40	0.40	0.40	0.40	0.40
				MRD	0.00	0.00	0.04	−0.05	−0.04	0.00	−0.06	0.01	−0.03
				Cor	1.00	0.99	0.98	0.92	0.92	0.92	0.92	0.92	0.92
High	Same (100%)	MD	0.00	0.00	−0.20	−0.02	0.00	−0.01	−0.01	0.01	−0.01
				MAD	0.25	0.09	0.20	0.49	0.49	0.48	0.48	0.48	0.48
				RMSE	0.28	0.11	0.20	0.61	0.61	0.60	0.61	0.61	0.61
				MRD	0.00	0.03	−0.92	−0.06	0.02	−0.07	−0.02	0.03	−0.02
				Cor	1.00	0.98	0.94	0.79	0.79	0.80	0.79	0.79	0.79
		Mixed (100%)	MD	0.00	0.00	0.01	−0.02	0.00	−0.01	−0.02	0.01	−0.01
				MAD	0.11	0.09	0.03	0.27	0.27	0.26	0.27	0.27	0.27
				RMSE	0.14	0.12	0.03	0.36	0.35	0.35	0.35	0.36	0.35
				MRD	0.00	0.00	0.05	−0.09	0.00	−0.04	−0.06	0.02	−0.03
				Cor	1.00	0.98	0.98	0.94	0.94	0.94	0.94	0.94	0.94

*Note*: MAD = mean absolute deviation; Cor = correlation; MD = mean deviation; MRD = mean relative deviation; RMSE = root mean square error.

**Table 5:** Triplet MCMC: Parameter recovery results

	Keyed direction				Intertrait						Mean of
Slope	(convergence rate)	Criterion	Slope	Intercept	correlation	Trait 1	Trait 2	Trait 3	Trait 4	Trait 5	Traits 1–5
Low	Same (89%)	MD	0.00	0.00	−0.10	−0.01	−0.01	0.00	0.00	0.00	0.00
				MAD	0.07	0.05	0.10	0.53	0.53	0.53	0.52	0.52	0.52
				RMSE	0.09	0.06	0.11	0.66	0.66	0.66	0.65	0.65	0.66
				MRD	−0.01	−0.04	−0.44	0.01	−0.04	−0.03	−0.01	0.03	−0.01
				Cor	0.99	0.99	0.95	0.75	0.75	0.75	0.76	0.76	0.75
		Mixed (100%)	MD	0.00	0.00	−0.02	−0.01	−0.01	0.00	0.00	0.00	0.00
				MAD	0.06	0.05	0.04	0.42	0.43	0.44	0.43	0.44	0.43
				RMSE	0.08	0.06	0.04	0.53	0.54	0.55	0.54	0.56	0.54
				MRD	0.00	0.00	−0.06	−0.02	0.01	0.04	0.00	−0.02	0.00
				Cor	0.99	0.99	0.96	0.85	0.84	0.84	0.84	0.83	0.84
Medium	Same (74%)	MD	−0.01	0.00	−0.10	0.01	−0.01	0.01	0.00	0.00	0.00
				MAD	0.11	0.06	0.11	0.49	0.47	0.48	0.47	0.47	0.48
				RMSE	0.13	0.08	0.11	0.61	0.59	0.60	0.59	0.60	0.60
				MRD	−0.01	0.02	−0.50	0.05	−0.02	0.02	0.01	0.01	0.01
				Cor	1.00	0.99	0.97	0.79	0.81	0.80	0.80	0.80	0.80
		Mixed (100%)	MD	0.00	0.00	−0.01	0.01	0.00	0.01	0.00	0.00	0.00
				MAD	0.08	0.07	0.03	0.31	0.31	0.31	0.31	0.31	0.31
				RMSE	0.10	0.09	0.03	0.39	0.39	0.39	0.39	0.40	0.39
				MRD	0.00	0.01	−0.03	−0.02	−0.01	0.03	0.01	−0.02	0.00
				Cor	1.00	0.99	0.98	0.92	0.92	0.92	0.92	0.92	0.92
High	Same (89%)	MD	0.00	0.00	−0.14	−0.01	−0.01	−0.01	0.00	0.00	−0.01
				MAD	0.19	0.08	0.14	0.48	0.48	0.47	0.48	0.48	0.48
				RMSE	0.22	0.10	0.15	0.61	0.60	0.60	0.60	0.60	0.60
				MRD	0.00	0.00	−0.64	0.00	0.00	−0.08	0.01	0.01	−0.01
				Cor	1.00	0.98	0.95	0.80	0.80	0.80	0.80	0.80	0.80
		Mixed (94%)	MD	0.00	0.00	−0.01	−0.01	0.00	−0.01	−0.01	0.00	−0.01
				MAD	0.11	0.09	0.03	0.26	0.26	0.26	0.26	0.26	0.26
				RMSE	0.13	0.12	0.03	0.33	0.34	0.33	0.33	0.34	0.33
				MRD	0.00	−0.01	−0.02	−0.05	0.00	−0.04	−0.01	−0.02	−0.02
				Cor	1.00	0.97	0.98	0.94	0.94	0.94	0.94	0.94	0.94

*Note*: MAD = mean absolute deviation; Cor = correlation; MD = mean deviation; MRD = mean relative deviation; RMSE = root mean square error.

Tables 4 and 5 show that the parameter estimates from the MML-EM and MCMC methods were very similar. Some general patterns in the simulation results from both estimation methods are observed as follows.

1. Across all conditions, the slopes and intercepts recovered well, as the correlations with the true values were larger than 0.97, MDs and MRDs were close to 0, and MAD and RMSE were small. The slopes in the same-direction-high-slope condition performed slightly worse, as their MADs and RMSEs were the largest (in the range from 0.19 to 0.28).
2. Across all conditions, the intertrait correlation estimates showed correlations with true values of greater than 0.94. However, the other four criteria in the same-direction conditions were worse than those in the mixed-direction conditions. Thus, the mixed-direction conditions had better intertrait correlation estimates than the same-direction conditions. The MCMC estimations show that the mean biases of the intertrait correlation estimates were all negative in all conditions; however, in the same-direction conditions, the mean biases were around −0.1, while in the mixed-direction conditions, they were around −0.01, indicating that trait score estimates in the mixed-direction conditions were better than the same-direction conditions. The mean biases of the intertrait correlation estimates from the MML-EM estimations were similar in magnitude to those of the MCMC; however, they had a positive direction, except in the same-direction conditions with medium or high slopes. In the low-slope-same-direction condition, the MML-EM method tended to overestimate intertrait correlations, while the MCMC method tended to underestimate them.
3. For the trait score estimates, the mixed-direction conditions showed moderate improvement over the same-direction conditions, with the percentage of increase in average correlations with true values ranging from 12% to 18%. With the mixed direction, the medium-slope condition increased the average correlation in the low-slope condition from 0.83 (or 0.84 for the MCMC method) to 0.92, while the increase from the medium-slope condition to the high-slope condition was only 0.02.

The MCMC estimations took much longer than the MML-EM ones. Because our simulation runs were conducted on different computers with varying hardware configurations and operating systems, we were unable to provide an overall summary of running times for both methods. However, we ran both MML-EM and MCMC estimations on 10 datasets in the medium-slope condition on a single computer with an Intel i7-4770U CPU, 16 GB of RAM, and Windows 10 OS. Based on the 10 datasets, the MML-EM estimations took an average of 37 and 38 min for the same- and mixed-direction conditions, respectively. In contrast, the average times for the MCMC estimations were 584 and 258 min, respectively.^2^

#### 6.3.2 Tetrads

Table 6 lists the parameter recovery results for the MML-EM estimations of tetrads, including the convergence rate and running time in each condition. Like triplets, only three runs in the same direction and one in the mixed direction under the low-slope condition were not converged. The parameter recovery for tetrads was similar or slightly better than that for triplets and showed similar patterns across conditions; moderate improvements were observed in latent trait score estimates in the low-slope conditions or the same-direction conditions, where the correlations with true values for tetrads increased, on average, from 0.04 to 0.07 compared to those for triplets. The average running time on a server with Intel Xeon E5-2640 v3 CPU, 256 GB of RAM, and Microsoft Windows Server 2016 Standard OS based on 21–23 converged runs^3^ was around 180 min, much longer than the average running times for triplets reported previously.

**Table 6:** Tetrad MML-EM: Parameter recovery results

	Keyed direction										
	(convergence rate;										
	mean running				Intertrait						Mean of
Slope	time [min]^a^)	Criterion	Slope	Intercept	correlation	Trait 1	Trait 2	Trait 3	Trait 4	Trait 5	Traits 1–5
Low	Same (91%; 174)	MD	0.00	0.00	0.04	−0.01	0.00	0.00	−0.01	0.02	0.00
				MAD	0.06	0.04	0.06	0.47	0.44	0.44	0.46	0.44	0.45
				RMSE	0.07	0.05	0.07	0.59	0.56	0.56	0.58	0.56	0.57
				MRD	0.01	0.01	0.21	−0.04	0.00	−0.01	−0.01	0.05	0.00
				Cor	1.00	1.00	0.97	0.81	0.83	0.83	0.81	0.83	0.82
		Mixed (97%; 174)	MD	0.00	0.00	0.01	−0.01	0.00	−0.01	−0.02	0.02	−0.01
				MAD	0.05	0.04	0.03	0.37	0.36	0.36	0.38	0.37	0.37
				RMSE	0.06	0.06	0.04	0.47	0.46	0.46	0.48	0.47	0.47
				MRD	0.00	−0.05	0.02	−0.01	−0.01	−0.01	−0.04	0.08	0.00
				Cor	1.00	0.99	0.97	0.88	0.89	0.89	0.88	0.89	0.89
Medium	Same (100%; 179)	MD	−0.01	−0.01	−0.09	−0.01	0.01	0.00	−0.01	0.02	0.00
				MAD	0.09	0.06	0.10	0.43	0.42	0.42	0.42	0.42	0.42
				RMSE	0.11	0.07	0.10	0.55	0.53	0.53	0.54	0.53	0.53
				MRD	0.00	−0.09	−0.46	−0.04	0.01	−0.04	−0.10	0.08	−0.02
				Cor	1.00	0.99	0.97	0.84	0.85	0.85	0.84	0.85	0.84
		Mixed (100%; 179)	MD	0.00	0.00	0.00	−0.01	0.01	0.00	−0.02	0.01	0.00
				MAD	0.07	0.06	0.03	0.27	0.26	0.26	0.27	0.27	0.27
				RMSE	0.08	0.08	0.03	0.35	0.34	0.34	0.35	0.35	0.34
				MRD	0.00	−0.02	0.01	−0.03	0.02	−0.06	−0.07	0.03	−0.02
				Cor	1.00	0.99	0.98	0.94	0.94	0.94	0.94	0.94	0.94
High	Same (100%; 185)	MD	−0.02	−0.01	−0.21	−0.02	0.00	−0.01	−0.01	0.00	−0.01
				MAD	0.26	0.07	0.21	0.44	0.44	0.44	0.45	0.45	0.44
				RMSE	0.29	0.09	0.22	0.56	0.56	0.56	0.57	0.57	0.56
				MRD	−0.01	0.00	−1.03	−0.09	0.02	−0.02	−0.04	−0.01	−0.03
				Cor	1.00	0.98	0.96	0.83	0.83	0.83	0.82	0.82	0.83
		Mixed (100%; 189)	MD	0.01	0.00	0.00	−0.02	−0.01	−0.01	−0.02	0.00	−0.01
				MAD	0.09	0.09	0.03	0.24	0.24	0.23	0.24	0.24	0.24
				RMSE	0.12	0.11	0.03	0.31	0.32	0.31	0.32	0.32	0.32
				MRD	0.00	0.07	−0.01	−0.10	−0.02	−0.04	−0.09	−0.02	−0.05
				Cor	1.00	0.98	0.98	0.96	0.96	0.96	0.95	0.95	0.95

*Note*: MAD = mean absolute deviation; Cor = correlation; MD = mean deviation; MRD = mean relative deviation; RMSE = root mean square error.

a Mean running time is based on 23 runs except for the low-slope-same-direction (21 runs) and low-slope-mixed-direction (22 runs) conditions on a server with Intel Xeon E5-2640 v3 CPU, 256 GB RAM, and Microsoft Windows Server 2016 Standard OS.

### 6.4 Results: Model comparison

The simulated Triplet data based on MGPPM were also estimated by the TIRT using the ULSMV method and by Triplet-2PLM using the MML-EM method. Their model fits and trait score estimations were compared to those of the MGPPM using the MML-EM method. All model and trait score estimations were converged in the TIRT and Triplet-2PLM.

Table 7 presents the summary statistics of the differences in Akaike information criterion (AIC) between Triplet-2PLM and MGPPM across converged replicates in both models for each of the six simulated conditions. Note that AIC differences are equal to the differences of Bayesian information criteria (BIC), and thus, Table 7 only shows the AIC differences. For AIC and BIC, smaller is better. A difference of at least 2 for AIC or 6 for BIC is considered strong evidence in favor of a better model (Bauldry, 2015). Table 7 shows that the MGPPM’s AIC and BIC were smaller than the Triplet-2PLM by at least 92 across all replicates, indicating that AIC and BIC prefer MGPPM over Triplet-2PLM. Note that Table 7 does not include the TIRT because the TIRT used a different dataset (i.e., recoded pair scores from triplet scores) from the other models (i.e., triplet scores), so their model comparison statistics, such as AIC and BIC, were incomparable.

**Table 7:** Comparisons of Triplet-2PLM and MGPPM on simulated triplet data: AIC difference (Triplet-2PLM–MGPPM)

Slope	Keyed direction	*n*	*Min*	*Max*	*M*	*SD*
Low	Same	33	92	260	166	36
		Mixed	34	132	242	181	33
Medium	Same	35	147	332	230	35
		Mixed	35	183	322	234	31
High	Same	35	184	321	251	31
		Mixed	35	165	312	230	31

*Note:* Triplet-2PLM and MGPPM were estimated by MML-EM. AIC difference is the same as BIC difference.

Table 8 presents the averages of the M2* Chi-square statistics (Cai & Hansen, 2013) and the related M2*-based RMSEA (root mean squared error of approximation) and SRMSR (standardized root mean squared residual) statistics for the three models across converged replicates under each condition. A *p*-value of M2* larger than a significant level (e.g., 0.01) indicates an overall good model fit. While M2* provides a significant test for omnibus model fit, RMSEA can be used to assess the goodness of approximation between the target model and the true model, and SRMSR indicates the magnitude of the misfit (the effect size). Maydeu-Olivares and Joe (2014; Table 2) suggested an RMSEA and SRMSR smaller than 0.05/ 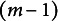 and 0.027/ 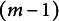 , respectively, for an excellent fit, smaller than 0.05 and 0.027, respectively, for a close fit, and smaller than 0.0089 and 0.05, respectively, for an adequate fit, where 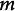 is the number of an item’s response categories (i.e., six for triplets). Table 8 shows that in all conditions, TIRT’s average M2* values were much larger than Triplet-2PLM’s and MGPPM’s, and TIRT’s mean p-values of the M2* tests were significant at 0.00, while the other two models’ were nonsignificant. Furthermore, TIRT’s mean RMSEA and SRMSR values were much larger than Triplet-2PLM’s and MGPPM’s, while Triplet-2PLM and MGPPM had similar values. TIRT’s mean RMSEA and SRMSR values decreased with higher item slopes, while the other two models’ values changed little across conditions. In the high-slope condition, TIRT’s mean SRMSR was just slightly higher than the other two models’. In general, the RMSEA values indicated close fits for TIRT and excellent fits for Triplet-2PLM and MGPPM; the SRMSR values indicated adequate fits for TIRT and close fits for Triplet-2PLM and MGPPM.

**Table 8:** Comparison of TIRT, Triplet-2PLM, and MGPPM on simulated triplet data: M2*, RMSEA, and SRMSR

Slope	Keyed direction	Model	M2*	*df*	*p*	*RMSEA*	*SRMSR*
Low	Same	TIRT	6030	1640	0.00	0.052	0.052
				Triplet–2PLM	108	100	0.35	0.008	0.031
				MGPPM	111	100	0.31	0.009	0.029
		Mixed	TIRT	5831	1640	0.00	0.051	0.050
				Triplet–2PLM	106	100	0.42	0.006	0.030
				MGPPM	109	100	0.35	0.008	0.028
Medium	Same	TIRT	4608	1640	0.00	0.043	0.038
				Triplet–2PLM	102	100	0.47	0.005	0.028
				MGPPM	107	100	0.37	0.007	0.025
		Mixed	TIRT	4311	1640	0.00	0.040	0.036
				Triplet–2PLM	103	100	0.43	0.006	0.027
				MGPPM	108	100	0.34	0.008	0.025
High	Same	TIRT	3498	1640	0.00	0.034	0.030
				Triplet–2PLM	105	100	0.42	0.006	0.027
				MGPPM	113	100	0.28	0.010	0.024
		Mixed	TIRT	3251	1640	0.00	0.031	0.029
				Triplet–2PLM	103	100	0.42	0.006	0.025
				MGPPM	109	100	0.31	0.009	0.024

*Note*: TIRT was estimated by ULSMV, and Triplet-2PLM was estimated by MML-EM. RMSEA = root mean squared error of approximation; SRMSR = standardized root mean squared residual.

**Table 9:** Comparisons of TIRT, Triplet-2PLM, and MGPPM on simulated triplet data: Reliability and correlation of latent score estimates

Slope	Keyed direction	Model	Stat	Trait 1	Trait 2	Trait 3	Trait 4	Trait 5	*M*
Low	Same	TIRT	Rel_ture	0.56	0.56	0.56	0.57	0.57	0.56
						Rel_est	0.65	0.65	0.65	0.65	0.65	0.65
						Cor_MGPPM	0.99	0.99	0.99	0.99	0.99	0.99
				Triplet –2PLM	Rel_ture	0.55	0.55	0.55	0.56	0.56	0.56
						Rel_est	0.58	0.58	0.57	0.57	0.56	0.57
						Cor_MGPPM	0.99	0.99	0.99	0.99	0.99	0.99
				MGPPM	Rel_ture	0.57	0.56	0.55	0.56	0.57	0.56
						Rel_est	0.57	0.57	0.56	0.57	0.56	0.57
		Mixed	TIRT	Rel_ture	0.72	0.70	0.70	0.71	0.69	0.70
						Rel_est	0.77	0.76	0.76	0.76	0.74	0.76
						Cor_MGPPM	1.00	1.00	1.00	1.00	1.00	1.00
				Triplet –2PLM	Rel_ture	0.71	0.70	0.70	0.70	0.68	0.70
						Rel_est	0.72	0.70	0.70	0.70	0.68	0.70
						Cor_MGPPM	1.00	1.00	1.00	0.99	0.99	1.00
				MGPPM	Rel_ture	0.72	0.70	0.70	0.71	0.69	0.70
						Rel_est	0.72	0.70	0.70	0.70	0.68	0.70
Medium	Same	TIRT	Rel_ture	0.62	0.65	0.64	0.64	0.64	0.64
						Rel_est	0.68	0.70	0.69	0.69	0.68	0.69
						Cor_MGPPM	0.99	0.99	0.99	0.99	0.99	0.99
				Triplet –2PLM	Rel_ture	0.62	0.65	0.63	0.64	0.64	0.63
						Rel_est	0.65	0.67	0.66	0.67	0.67	0.67
						Cor_MGPPM	0.99	0.99	0.99	0.99	1.00	0.99
				MGPPM	Rel_ture	0.63	0.65	0.64	0.65	0.64	0.64
						Rel_est	0.63	0.66	0.65	0.66	0.66	0.65
		Mixed	TIRT	Rel_ture	0.85	0.85	0.85	0.85	0.84	0.85
						Rel_est	0.87	0.87	0.87	0.87	0.86	0.87
						Cor_MGPPM	1.00	1.00	1.00	1.00	1.00	1.00
				Triplet –2PLM	Rel_ture	0.84	0.85	0.85	0.85	0.84	0.84
						Rel_est	0.84	0.85	0.84	0.84	0.84	0.84
						Cor_MGPPM	1.00	1.00	1.00	1.00	1.00	1.00
				MGPPM	Rel_ture	0.85	0.85	0.85	0.85	0.84	0.85
						Rel_est	0.84	0.84	0.84	0.84	0.83	0.84
High	Same	TIRT	Rel_ture	0.62	0.62	0.63	0.63	0.62	0.63
						Rel_est	0.69	0.68	0.69	0.68	0.67	0.68
						Cor_MGPPM	0.99	0.98	0.98	0.98	0.98	0.98
				Triplet –2PLM	Rel_ture	0.62	0.62	0.63	0.62	0.62	0.62
						Rel_est	0.68	0.70	0.70	0.71	0.72	0.70
						Cor_MGPPM	1.00	1.00	1.00	1.00	1.00	1.00
				MGPPM	Rel_ture	0.63	0.62	0.63	0.63	0.63	0.63
						Rel_est	0.67	0.68	0.69	0.69	0.71	0.69
		Mixed	TIRT	Rel_ture	0.89	0.89	0.89	0.89	0.88	0.89
						Rel_est	0.89	0.89	0.90	0.89	0.89	0.89
						Cor_MGPPM	1.00	1.00	1.00	1.00	1.00	1.00
				Triplet –2PLM	Rel_ture	0.89	0.89	0.89	0.89	0.88	0.89
						Rel_est	0.88	0.88	0.88	0.88	0.88	0.88
						Cor_MGPPM	1.00	1.00	1.00	1.00	1.00	1.00
				MGPPM	Rel_ture	0.89	0.89	0.89	0.89	0.88	0.89
						Rel_est	0.88	0.87	0.88	0.88	0.87	0.87

*Note:* TIRT was estimated by ULSMV, and Triplet-2PLM and MGPPM were estimated by MML-EM. Cor_MGPPM = Correlation of latent score estimates with MGPPM’s; Rel_ture = Squared correlation between true and estimated latent scores (true reliability); Rel_est = Estimated reliability of latent score estimates (based on Equation 40).

The recovery of trait score estimations for TIRT and Triplet-2PLM was very similar to MGPPM’s. The evaluation results for TIRT and Triplet-2PLM, based on the five criteria in Table 4, are presented in Table A1 in the SOM. Table 9 shows the average true reliabilities, estimated reliabilities, and correlations with MGPPM’s trait score estimates for all three estimation methods and five traits across the converged runs in each condition. True reliability is defined as the squared correlation between true and estimated trait scores. The empirical reliability of a trait’s MAP score estimates is estimated as follows (Kim, 2012): 
(40)

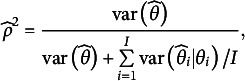

where 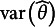 is the sample variance of trait score estimates, 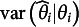 is the error variance of subject 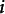 ’s estimated trait score 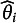 , and 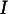 is the sample size. In Table 9, the average correlations of TIRT’s and Triplet-2PLM’s trait score estimates with MGPPM’s were at least 0.98, and their trait score reliabilities were very similar in each condition. One exception is that in the low-slope condition, TIRT’s estimated reliabilities were larger than those of the other two models and inflated compared to the true reliabilities. The inflation of estimated reliabilities was also observed in the medium-slope-same-direction condition for TIRT and Triplet-2PLM, as well as in the high-slope-same-direction condition for all three methods. Theoretically, for TIRT, due to the local independence assumption in trait score estimation, redundant information is added to the test information, resulting in an overestimated reliability (Frick, 2023). However, our result showed that reliability overestimation occurred for TIRT only when the true reliability was low (e.g., smaller than 0.8), and other factors also impacted reliability estimation. In addition, under certain conditions, Triplet-2PLM and MGPPM also overestimate score reliabilities. Future research is needed to further investigate this issue.

In terms of estimation times, a TIRT estimation in Mplus, without estimating standard errors, completed in 1–2 s on average on a laptop with an Intel Core i5-1345U CPU and 16GB RAM, while a typical Triplet-2PLM or MGPPM run in R took from about 39 to 53 min on a Linux server. In general, an MGPPM run was slightly faster than a Triplet-2PLM one; however, MGPPM’s running times were more divergent than Triplet-2PLM’s. Table A2 in the SOM summarizes the running items of the three models in each condition.

In summary, the simulation results showed that when estimating simulated data generated from MGPPM, although the fit statistics might prefer the generating model over the TIRT and Triplet-2PLM, the trait score estimates from the three models were practically indifferent.

## 7 Real data application

The real data were from a triplet form that measured 14 interpersonal and intrapersonal skills essential to higher education and career success, such as perseverance, leadership, creativity, curiosity, responsibility, and self-discipline. The triplet form consisted of 60 blocks, totaling 180 dominance statements. Each statement in an item measured a different trait, and each trait was measured by 5–18 statements. There were 25 items with three negative statements, 30 with three positive statements, one with one positive and two negative statements, and four with two positive and one negative statements.^4^ The valid sample size is 538 without any missing responses. Among the 538 test takers, 258 were recruited from the crowdsourcing website Amazon Mechanical Turk (AMT), and 280 were applicants to a university. Among the 258 AMT samples, 43% were male, and 57% were female; the ethnicity distribution was 75% White, 12% Black, 7% Asian, and 7% Hispanic; the distribution of education experience was 5% high-school diploma, 16% associate’s degree, 57% bachelor’s degree, and 22% postgraduate degree.

We applied the MGPPM, TIRT, and Triplet-2PLM to the real data. For all models, the intertrait correlation matrix was fixed to that in Table 3 during the estimations to stabilize the estimates due to the high dimension (14 traits) of the real data. Table 3 was obtained from previous administrations of the Likert scales of the statements. We used the MAP trait score estimates for all models. The program setups for estimating the three models were identical to those used in the simulation study.

Below, we compared the results from the three methods regarding model selection, overall model fit, item fit, local dependence test, and trait score reliability and correlation.

### 7.1 Results

Table 10 shows the model comparison result between the Triplet-2PLM and MGPPM based on the log likelihood, Akaike information criterion (AIC), and Bayesian information criteria (BIC). In Table 10, the MGPPM’s AIC and BIC were 253 smaller than the Triplet-2PLM’s, indicating a better fit.

**Table 10:** Comparison of log likelihood, AIC, and BIC

Model	logLik	Number of item parameters	*AIC*	AIC_dif^a^	*BIC*	BIC_dif^a^
Triplet–2PLM	−53,110	300	106,819		108,105	
MGPPM	−52,983	300	106,567	253	107,853	253

a Model above – Model below.

Table 11 presents the M2*, RMSEA, and SRMSR statistics for the three models. The M2* statistics were significant for all models, and the SRMSR statistics were nearly identical across all models, exceeding the threshold for an adequate fit (0.5). In contrast, MGPPM’s and Triplet-2PLM’s RMSEA statistics were slightly smaller than TIRT’s, and they all were smaller than 0.05, indicating all models had close but perfect fits to the data.

**Table 11:** Comparison of M2* and RMSEA

Model	M2*	*df*	*p*	*RMSEA*	*SRMSR*
TIRT	33,030	15,990	0.00	0.045	0.064
Triplet–2PLM	2731	1530	0.00	0.038	0.064
MGPPM	2738	1530	0.00	0.036	0.063

*Note*: RMSEA = root mean squared error of approximation; SRMSR = standardized root mean squared residual.

We use the 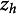 statistic (Drasgow et al., 1985) to assess the fit of an individual item. Note that items in the TIRT model refer to the pairs stemming from the triplets. The asymptotic distribution of the 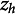 statistic is standard normal, assuming true model parameters. The hypothesis test is conducted structurally as a lower one-sided test. Because the estimated model parameters from these IRT models were used in estimating 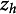 , the asymptotic distributions of the 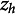 statistics were unknown. However, we used 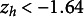 for the Triplet-2PLM and MGPPM and 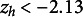 for the TIRT model (due to the Bonferroni adjustment) as the rough criteria to flag potential misfit items corresponding to a one-sided significance level of 0.05 based on the standard normal distribution. Table 12 shows only one misfitted pair (stemming from a triplet) in the TIRT model and none in the other two models.

**Table 12:** Comparison of number (*N*) and percentage (%) of misfit items

	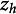 ^a^		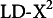 ^b^	
Model	N	%	N	%
TIRT	1	1	58	0
Triplet–2PLM	0	0	0	0
MGPPM	0	0	0	0

a Flagging criterion 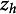 < −1.64 for the Triple-2PLM and MGPPM and 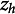 < −2.13 for the TIRT model.

b Flagging criterion Cramer’s *V* > 0.3.

An item pair fit test has been used to check the local independence assumption in IRT models. We used Chen and Thissen’s (1997) Pearson 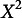 local dependence ( 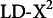 ) index to check the fits of item pairs. Because the asymptotic distribution of 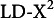 is unknown, we transformed the 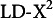 index to Cramer’s V index (Cramer, 1946, p. 282), a normalized version of the 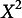 test statistic, and used the criterion, Cramer’s *V* larger than 0.3, as a rough indicator of strong local dependence among items. The justification for applying the 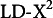 test to the TIRT models lies in the local independence assumption among the three pairs that stem from a triplet in trait score estimation. In Table 12, a tiny (0.36%) proportion of item pairs (i.e., 58 out of 16,110 pairs of items) in the TIRT model showed strong local dependence, while none did for the other two models.

Table 13 shows that the Triplet-2PLM and MGPPM had almost identical reliability estimates across the 14 traits, and both had the mean and SD of the reliability estimates of 0.68 and 0.07, respectively. In contrast, the TIRT model had slightly and consistently higher reliability estimates than the other two models across the 14 traits; the mean and SD of the reliability estimates were 0.74 and 0.06, respectively, for the TIRT model. The reliability estimates in the TIRT model were likely inflated due to the local independence assumption among the pairs stemming from the triplets in trait score estimations. This result is consistent with that in the low-slope condition of the previous simulation study, as the slope’s distribution parameters in the low-slope condition were derived from real data.

**Table 13:** Comparison of trait score reliability estimates

Trait	1	2	3	4	5	6	7	8	9	10	11	12	13	14	*M*	*SD*
TIRT	0.71	0.80	0.68	0.74	0.66	0.78	0.70	0.78	0.86	0.69	0.72	0.80	0.72	0.74	0.74	0.06
Triplet- 2PLM	0.61	0.75	0.57	0.65	0.58	0.74	0.65	0.72	0.82	0.62	0.68	0.76	0.68	0.69	0.68	0.07
MGPPM	0.60	0.74	0.57	0.66	0.58	0.73	0.65	0.71	0.81	0.61	0.68	0.76	0.68	0.68	0.68	0.07

Table 14 lists the correlations of trait score estimates between the MGPPM and the other two models. The Triplet-2PLM showed nearly perfect correlations with the MGPPM, with a mean correlation of 0.99 and a standard deviation of 0.00. The TIRT’s correlations were slightly lower, with a mean and standard deviation of 0.98 and 0.01, respectively. This result is also consistent with that in the simulation study.

**Table 14:** Correlations of trait score estimates with MGPPM

Trait	1	2	3	4	5	6	7	8	9	10	11	12	13	14	*M*	*SD*
TIRT	0.97	0.98	0.96	0.99	0.96	0.98	0.96	0.99	0.99	0.95	0.98	0.99	0.98	0.98	0.98	0.01
Triplet- 2PLM	0.99	0.99	0.98	0.99	0.99	0.99	0.99	1.00	1.00	0.99	0.99	0.99	0.99	0.99	0.99	0.00

In summary, all three models provided a good fit to the real data, as indicated by the model and item fit statistics. Their trait score estimates were almost perfectly correlated. The Triplet-2PLM and MGPPM had almost identical reliability estimates on trait scores, while the TIRT model’s reliability estimates were slightly inflated. Based on the model comparison statistics, the MGPPM fitted the data better than the Triplet-2PLM.

## 8 Discussion

We have proposed a new approach, the ranking pattern approach, to build IRT models for FC items, which is a substantial addition to the two existing approaches, sequential selection and Thurstone’s law of pairwise comparison. The current study makes a valuable contribution to the psychometric literature, offering both theoretical and practical insights.

Based on the ranking pattern approach, we have built a new dominance IRT model, MGPPM, and developed its MML-EM and MCMC estimations. We conducted a simulation study on triplets and tetrads to inspect their parameter recovery under various slope and keyed-direction conditions using the MML-EM and/or MCMC estimations. Both estimation methods generated nearly identical estimates for triplets; however, the MCMC estimations required substantially more running time than the MML-EM estimations. The slope and intercept estimations performed well under all conditions, while the intertrait correlations were estimated better in the mixed-direction conditions than in the same-direction conditions. Furthermore, the mixed-direction conditions moderately improved the trait score estimations compared to the same-direction conditions. Within the mixed-direction conditions, the medium slopes showed moderate improvement in trait score estimations compared to the low slopes. In contrast, the improvement from the medium- to high-slope conditions was minor. Generally, the medium-slope, mixed-direction condition yielded the best model estimations. These results on triplets were generally consistent with the findings in the simulation study for the Triplet-2PLM in Fu et al. (2025). The results on tetrads were similar to or slightly better than those on triplets, and moderate improvements compared to triplets were observed in latent trait score estimates in the low-slope conditions or the same-direction conditions.

We have applied the three models, TIRT, Triplet-2PLM, and MGPPM, to the triplet data simulated based on MGPPM and compared their model fits and trait score estimates. The model comparison statistics, AIC and BIC, indicated that MGPPM was a better-fitted model than Triplet-2PLM. In contrast, the model fit statistics, M2*, RMSEA, and SRMSR, showed that the two models performed similarly and fitted the simulated data better than TIRT. Nevertheless, the trait score estimations of the three models were almost identical. We also applied the three models to a real triplet dataset and compared their results. All model and item fit statistics showed that all three models fitted the real data well, except that the AIC and BIC statistics preferred MGPPM over Triplet-2PLM. Like in the simulation study, their trait score estimates were nearly perfectly correlated. Both the simulation and real studies showed that, when a true reliability was low in a test, the estimated reliability in the TIRT model was spuriously high due to the local independence assumption among pairs stemming from a triplet in trait score estimations.

We have provided a theoretical and insightful comparison among the three approaches/models for FC items. The Triplet-2PLM and MGPPM are simpler models than the TIRT model because they simplify the joint distribution of a ranking pattern by making assumptions and have fewer model parameters. The MGPPM has more concise and straightforward conditional logit functions than the Rank-2PLM and TIRT, and thus, it is a well-defined model. In addition, the ranking pattern approach provides a more flexible and straightforward way to incorporate various assumptions regarding a ranking process into IRT models than the other two approaches, and it can handle scoring schemes for new item formats that the other two approaches cannot. The ranking pattern approach has this advantage because its basic modeling unit is the utility of a statement with a rank. In contrast, the other two approaches’ basic modeling unit is the utility of a statement regardless of its rank. Also, because of this modeling difference, unlike a model in the other two approaches, a model in the ranking pattern approach is not a function of the response functions of individual statements for items with more than two statements; thus, its parameters do not correspond to the parameters of the response functions of individual statements. This feature has implications for FCQ form assemblies and administrations.

A common practice for assembly and administration of an FC test form is (a) pretesting the statements in Likert-type forms, (b) calibrating the Likert tests based on 2PLM or GGUM, (c) assembling FC forms based on item parameters on each statement, and (d) administering the MFC forms and score them by an IRT model for FCQs with fixed item parameters from the Likert tests. This practice enables the automatic generation of multiple FC forms by a test assembly program, such as those used in computer adaptive testing (CAT) programs, and provides instant score reports to test takers. Although it is fine to use item parameters from Likert scales to assist initial form building, it raises many questions regarding using the Likert item parameters in the IRT scoring of FC items because this practice ignores the potential differences in item types (Likert vs. FC), item context (i.e., a statement may have different performance when matching with different statements in an FC item), or test sample (i.e., the sample taking the Likert test may be different from that taking the FCQ); see Fu, Kyllonen, and Tan (2024a) for a detailed study of this issue. A remedy proposed is to use the TIRT or Rank models to estimate item parameters directly from FC data, either from a field test or accumulated from initial operational testing. Then, use the directly estimated item parameters to score FCQs and update the item parameters periodically based on newly collected data, if needed. However, unless the context-free on FC items is assumed, these statement parameters are still item-dependent and cannot be used across items, which seriously limits the capacity of a CAT administration. If the MGPPM is used for an FCQ testing program, all statement parameters are item-dependent and estimated directly from FC data, meaning all FC items must be field-tested. Thus, the MGPPM is only suitable for a testing program with a limited number of fixed forms.

### 8.1 Limitations and future research

The current study has several limitations, and we suggest that further studies be conducted. First, the simulation conditions considered in the current study were not comprehensive. A more comprehensive simulation study with a wider range of conditions would be worthwhile for both parameter recovery and model comparisons. For example, for model comparisons, we can generate data based on each of the three models rather than just MGPPM and then estimate the simulated data using all three models. Second, the item fit statistics ( 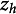 and 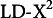 ) used in the real data study are theoretically appropriate for these models; however, their performance on these models has not yet been systematically studied. Future research is needed to investigate item fit statistics on these and other IRT models for FC items. Third, we did not delve into the mathematical details to explain why the mixed-direction conditions performed better than the same-direction conditions in intertrait correlation and trait score estimations. This issue has been explored in, for example, Brown and Maydeu-Olivares (2011) and Bürkner (2022) for the TIRT model. Investigating this issue on the MGPPM will also be interesting, as it will provide more insights into the model. Finally, as we mentioned previously, the ranking pattern approach offers a more flexible IRT modeling than the other two approaches; however, we only studied one model, MGPPM, in this paper. More models using this approach can be proposed and investigated to meet practical needs.

## Supporting information

Furr and Fu supplementary material Furr and Fu supplementary material

## Data Availability

The real dataset used in the current study is not publicly available, as it is the property of Educational Testing Service. However, it is available from the corresponding author upon reasonable request.

## Footnotes

1
Brown and Maydeu-Olivares (2011) ignored this constraint and adjusted the model’s degrees of freedom (DF) after estimation. Yousfi (2025) has argued that this constraint is necessary for the TIRT model to avoid intransitive pairwise comparisons. Our experience is that whether imposing this constraint or not seems practically unimportant. Either way, the threshold estimates are very close because the data do not contain intransitive pairwise comparisons. Once this constraint is imposed, there is no need to adjust DF after estimation.

2
For the MCMC runs, we used the option of parallel processing in *rstan*, so the four chains ran simultaneously. However, the parallel processing option in *mirt* did not improve the running times much, and it caused a running error in our program. Thus, we did not use parallel processing in the MML-EM runs. For an MCMC run, we reported the running time as the longest running time among the four chains.

3
Other runs were completed on multiple Linux servers.

4
For more information regarding the development of this triplet form, refer to Fu, Kyllonen, and Tan (2024, Form 3B).

## Supplementary material

To view supplementary material for this article, please visit http://doi.org/10.1017/psy.2025.10054.

## References

[r1] Andrich, D. (1995). Hyperbolic cosine latent trait models for unfolding direct-responses and pairwise preferences. Applied Psychological Measurement, 19(3), 269–290. 10.1177/014662169501900306

[r2] Atkinson, K. E. (1989). An introduction to numerical analysis (2^nd^ ed.). John Wiley.

[r3] Baker, F. B. , & Kim, S. H. (2004). Item response theory: Parameter estimation techniques (2^nd^ ed.). CRC Press. 10.1201/9781482276725

[r4] Bauldry, S. (2015). Structural equation modeling. In D. W. James (Ed.), International Encyclopedia of the Social & Behavioral Sciences (2^nd^ ed., pp. 615–620). Elsevier. 10.1016/B978-0-08-097086-8.44055-9

[r5] Birnbaum, A. (1968). Some latent trait models and their use in inferring an examinee’s ability. In F. M. Lord & M. R. Novick (Eds.), Statistical theories of mental test scores (pp. 397–479). Addison-Wesley.

[r6] Bock, R. D. , & Aitkin, M. (1981). Marginal maximum likelihood estimation of item parameters: Application of an EM algorithm. Psychometrika, 46(4), 443–459. 10.1007/BF02293801

[r7] Bradley, R. A. (1953). Some statistical methods in taste testing and quality evaluation. Biometrics, 9(1), 22–38. 10.2307/3001630

[r8] Brown, A. (2016). Item response models for forced-choice questionnaires: A common framework. Psychometrika, 81(4), 135–160. 10.1007/s11336-014-9434-9 25663304

[r9] Brown, A. , & Maydeu-Olivares, A. (2011). Item response modeling of forced-choice questionnaires. Educational and Psychological Measurement, 71(3), 460–502. 10.1177/0013164410375112

[r10] Brown, A. , & Maydeu-Olivares, A. (2018). Ordinal factor analysis of graded-preference questionnaire data. Structural Equation Modeling: A Multidisciplinary Journal, 25(4), 516–529. 10.1080/10705511.2017.1392247

[r11] Broyden, C. G. (1970). The convergence of a class of double-rank minimization algorithms 1. General considerations. Journal of Applied Mathematics, 6(1), 76–90. 10.1093/imamat/6.1.76

[r12] Bürkner, P.-C. , Schulte, N. , & Holling, H. (2019). On the statistical and practical limitations of Thurstonian IRT models. Educational and Psychological Measurement, 79(5), 827–854. 10.1177/0013164419832063 31488915 PMC6713979

[r13] Bürkner, P. C. (2022). On the information obtainable from comparative judgments. Psychometrika, 87(4), 1439–1472. 10.1007/s11336-022-09843-z 35133553 PMC9636126

[r14] Bürkner, P. C. (2024). *thurstonianIRT: Thurstonian IRT Models* (R package Version 0.12.5) [Computer software]. https://CRAN.R-project.org/package=thurstonianIRT

[r15] Cai, L. (2010). High-dimensional exploratory item factor analysis by a Metropolis-Hastings Robbins-Monro algorithm. Psychometrika, 75(1), 33–57. 10.1007/s11336-009-9136-x

[r16] Cai, L. , & Hansen, M. (2013). Limited-information goodness-of-fit testing of hierarchical item factor models. British Journal of Mathematical and Statistical Psychology, 66(2), 245–276. 10.1111/j.2044-8317.2012.02050.x 22642552 PMC3760206

[r17] Cao, M. , & Drasgow, F. (2019). Does forcing reduce faking? A meta-analytic review of forced-choice personality measures in high-stakes situations. Journal of Applied Psychology, 104(11), 1347–1368. 10.1037/apl0000414 31070382

[r18] Chalmers, R. (2012). Mirt: A multidimensional item response theory package for the R environment. Journal of Statistical Software, 48(6), 1–29. 10.18637/jss.v048.i06

[r19] Chen, W. H. , & Thissen, D. (1997). Local dependence indices for item pairs using item response theory. Journal of Educational and Behavioral Statistics, 22(3), 265–289. 10.2307/1165285

[r20] Christiansen, N. D. , Burns, G. N. , & Montgomery, G. E. (2005). Reconsidering forced-choice item formats for applicant personality assessment. Human Performance, 18(3), 267–307. 10.1207/s15327043hup1803_4

[r21] Clausen, A. , & Sokol, S. (2020). *Deriv: R-based Symbolic Differentiation* (R package Version 4.1) [Computer software]. https://CRAN.R-project.org/package=Deriv

[r22] Cramer, H. (1946). Mathematical methods of statistics. Princeton University Press.

[r23] Davis, P. J. , & Rabinowitz, P. (1984). Methods of numerical integration. Academic Press. 10.1016/B978-0-12-206360-2.50012-1

[r24] de la Torre, J. , Ponsoda, V. , Leenen, I. , & Hontangas, P. (2012). *Examining the viability of recent models for forced-choice data* [Paper presentation]. American Educational Research Association.

[r25] Drasgow, F. , Levine, M. V. , & Williams, E. A. (1985). Appropriateness measurement with polychotomous item response models and standardized indices. British Journal of Mathematical and Statistical Psychology, 38(1), 67–86. 10.1111/j.2044-8317.1985.tb00817.x

[r26] Figueiras, D. , Williams, K. M. , Fu, J. , Roohr, K. , Ling, G. , & Wang, Y. (2025). *Faking resistance in hybrid forced-choice Likert (HFCL) and Likert-scale personality assessment: A comparative study* [Manuscript submitted for publication]. Educational Testing Service.

[r27] Forero, C. G. , & Maydeu-Olivares, A. (2009). Estimation of IRT graded response models: Limited versus full information methods. Psychological Methods, 14(3), 275–299. 10.1037/a0015825 19719362

[r28] Frick, S. (2023). Estimating and using block information in the Thurstonian IRT model. Psychometrika, 88(4), 1556–1589. 10.1007/s11336-023-09931-8 37640828 PMC10656335

[r29] Fu, J. (2019). *Maximum marginal likelihood estimation with an expectation-maximization algorithm for multigroup/mixture multidimensional item response theory models*. (ETS Research Report No. 19-35). Educational Testing Service. 10.1002/ets2.12272

[r30] Fu, J. (2025). Maximum marginal likelihood estimation of the MUPP-GGUM model. Applied Psychological Measurement. 10.1177/01466216251336925 PMC12009269 40260227

[r31] Fu, J. , Kyllonen, P. C. , & Tan, X. (2024a). From Likert to forced choice: Statement parameter invariance and context effects in personality assessment. Measurement: Interdisciplinary Research and Perspective, 22(3), 280–296. 10.1080/15366367.2023.2258482 PMC12291097 40718505

[r32] Fu, J. , Tan, X. , & Kyllonen, P. C. (2024b). Item and test characteristic curves of rank-2PL models for multidimensional forced-choice questionnaires. Applied Measurement in Education, 37(3), 272–288. 10.1080/08957347.2024.2386939 40881481 PMC12385645

[r33] Fu, J. , Tan, X. , & Kyllonen, P. C. (2025). The rank-2PL IRT models for forced-choice questionnaires: Maximum marginal likelihood estimation with an EM algorithm. Journal of Educational and Behavioral Statistics, 50(3), 497–525. 10.3102/10769986241256030 40874117 PMC12379955

[r34] Fu, J. , Tan, X. , & Kyllonen, P. C. (in press). Can the generalized graded unfolding model fit dominance responses? *Applied Psychological Measurement*. 10.1177/01466216251401214 PMC12682685 41368044

[r35] Gilks, W. R. , Richardson, S. , & Spiegelhalter, D. J. (1996). Introducing Markov chain Monte Carlo. In W. R. Gilks , S. Richardson , & D. J. Spiegelhalter (Eds.), Markov chain Monte Carlo in practice (pp. 1–20). Chapman & Hall. 10.1201/b14835

[r36] Gelman, A. (1996). Inference and monitoring convergence. In W. R. Gilks , S. Richardson , & D. J. Spiegelhalter (Eds.), Markov chain Monte Carlo in practice (pp. 131–143). Chapman & Hall. 10.1201/b14835

[r37] Hoffman, M. D. , & Gelman, A. (2014). The no-U-turn sampler: Adaptively setting path lengths in Hamiltonian Monte Carlo. Journal of Machine Learning Research, 15(47), 1593–1623.

[r38] Hontangas, P. M. , de la Torre, J. , Ponsoda, V. , Leenen, I. , Morillo, D. , & Abad, F. J. (2015). Comparing traditional and IRT scoring of forced-choice tests. Applied Psychological Measurement, 39(8), 598–612. 10.1177/0146621615585851 29881030 PMC5978493

[r39] Joo, S.-H. , Lee, P. , & Stark, S. (2018). Development of information functions and indices for the GGUM-RANK multidimensional forced choice IRT model. Journal of Educational Measurement, 55(3), 357–372. 10.1111/jedm.12183

[r40] Kim, S. (2012). A note on the reliability coefficients for item response model-based ability estimates. Psychometrika, 77(4), 153–162. 10.1007/s11336-011-9238-0

[r41] Lee, P. , Joo, S. H. , Stark, S. , & Chernyshenko, O. S. (2019). GGUM-RANK statement and person parameter estimation with multidimensional forced choice triplets. Applied Psychological Measurement, 43(3), 226–240. 10.1177/0146621618768294 31019358 PMC6463341

[r42] Li, Z. , Li, L. , Zhang, B. , Cao, M. , & Tay, L. (2025). Killing two birds with one stone: Accounting for unfolding item response process and response styles using unfolding item response tree models. Multivariate Behavioral Research, 60(2), 161–183. 10.1080/00273171.2024.2394607 39215711

[r43] Luce, R. D. (1959). Individual choice behavior: A theoretical analysis. John Wiley.

[r44] Maydeu-Olivares, A. (1999). Thurstonian modeling of ranking data via mean and covariance structure analysis. Psychometrika, 64(3), 325–340. 10.1007/BF02294299

[r45] Maydeu-Olivares, A. , & Brown, A. (2010). Item response modeling of paired comparison and ranking data. Multivariate Behavioral Research, 45(6), 935–974. 10.1080/00273171.2010.531231 26760724

[r46] Maydeu-Olivares, A. , & Joe, H. (2014). Assessing approximate fit in categorical data analysis. Multivariate Behavioral Research, 49(4), 305–328. 10.1080/00273171.2014.911075 26765800

[r47] McDonald, R. P. (1997). Normal-Ogive multidimensional model. In W. J. van der Linden & R. K. Hambleton (Eds.), Handbook of modern item response theory (pp. 258–270). Springer.

[r48] McFadden, D. (1973). Conditional logit analysis of qualitative choice behavior. In P. Zarembka (Ed.), Frontiers in econometrics (pp. 105–142). Academic Press.

[r49] Morillo, D. , Leenen, I. , Abad, F. J. , Hontangas, P. , De la Torre, J. , & Ponsoda, V. (2016). A dominance variant under the multi-unidimensional pairwise-preference framework: Model formulation and Markov chain Monte Carlo estimation. Applied Psychological Measurement, 40(7), 500–516. 10.1177/0146621616662226 29881066 PMC5978637

[r50] Muraki, E. (1993). Information functions of the generalized partial credit model. Applied Psychological Measurement, 17(4), 351–363. 10.1177/014662169301700403

[r51] Muthén, L. K. , & Muthén, B. O. (1998–2017). Mplus user’s guide (8^th^ ed.). Muthén & Muthén.

[r52] R Core Team (2023). R: A language and environment for statistical computing (version 4.3.2). R Foundation for Statistical Computing. https://www.R-project.org/

[r53] Roberts, J. S. , Donoghue, J. R. , & Laughlin, J. E. (2000). A general item response theory model for unfolding unidimensional polytomous responses. Applied Psychological Measurement, 24(1), 3–32. 10.1177/01466216000241001

[r54] Samejima, F. (1969). Estimation of latent ability using a response pattern of graded scores. Psychometrika Monograph Supplement, 34(4, Pt. 2), 100.

[r55] Sass, R. , Frick, S. , Reips, U.-D. , & Wetzel, E. (2020). Taking the test taker’s perspective: Response process and test motivation in multidimensional forced-choice versus rating scale instruments. Assessment, 27(3), 572–584. 10.1177/1073191118762040 29560735

[r56] Schilling, S. , & Bock, R. D. (2005). High-dimensional maximum marginal likelihood item factor analysis by adaptive quadrature. Psychometrika, 70(3), 533–555. 10.1007/s11336-003-1141-x

[r57] Sisson, E. D. (1948). Forced choice: The new army rating. Personnel Psychology, 1(3), 365–381. 10.1111/j.1744-6570.1948.tb01316.x

[r58] Stan Development Team (2024). *RStan: the R interface to Stan* (Version 2.32.5). https://mc-stan.org/

[r59] Stark, S. , Chernyshenko, O. S. , & Drasgow, F. (2005). An IRT approach to constructing and scoring pairwise preference items involving stimuli on different dimensions: The multi-unidimensional pairwise preference model. Applied Psychological Measurement, 29(3), 184–203. 10.1177/0146621604273988

[r60] Thurstone, L. L. (1927). A law of comparative judgment. Psychological Review, 34(4), 273–286. 10.1037/h0070288

[r61] Thurstone, LL (1929). The measurement of psychological value. In T. V. Smith & W. K. Wright (Eds.), Essays in philosophy by seventeen doctors of philosophy of the University of Chicago (pp. 157–174). Open Court.

[r62] van der Ark, L. A. (2001). Relations and properties of polytomous item response theory models. Applied Psychological Measurement, 25(3), 273–283. 10.1177/01466210122032073

[r63] von Davier, M. (2008). A general diagnostic model applied to language testing data. British Journal of Mathematical and Statistical Psychology, 61(2), 287–307. 10.1348/000711007X193957 17535481

[r64] Wetzel, E. , Frick, S. , & Brown, A. (2021). Does multidimensional forced-choice prevent faking? Comparing the susceptibility of the multidimensional forced-choice format and the rating scale format to faking. Psychological Assessment, 33(2), 156–170. 10.1037/pas0000971 33151727

[r65] Williams, K. M. , Figueiras, D. , & Roohr, K. (2025). *Addressing user experience concerns of forced-choice: Introducing a hybrid forced-choice/Likert (HFCL) response format* [Manuscript submitted for publication]. Educational Testing Service.

[r66] Yousfi, S. (2025). Statistical foundations of person parameter estimation in the Thurstonian IRT model for forced-choice and pairwise comparison designs. British Journal of Mathematical and Statistical Psychology, 78(2), 555–593. 10.1111/bmsp.12364 39601492

[r67] Zhang, B. , Luo, J. , & Li, J. (2023). Moving beyond Likert and traditional forced-choice scales: A comprehensive investigation of the graded forced-choice format. Multivariate Behavioral Research. 10.1080/00273171.2023.2235682 37652572

[r68] Zhang, B. , Tu, N. , Angrave, L. , Zhang, S. , Sun, T. , Tay, L. , & Li, J. (2024). The generalized Thurstonian unfolding model (GTUM): Advancing the modeling of forced-choice data. Organizational Research Methods, 27(4), 713–747. 10.1177/10944281231210481

[r69] Zheng, C. , Liu, J. , Li, Y. , Xu, P. , Zhang, B. , Wei, R. , Zhang, W. , Liu, B. , & Huang, J. (2024). A 2PLM-RANK multidimensional forced-choice model and its fast estimation algorithm. Behavior Research Methods, 56, 6363–6388. 10.3758/s13428-023-02315-x 38409459

