# Supplementary material for: Multidimensional Generalized Partial Preference Model for Forced-Choice Items
Source: Psychometrika. 2025 Nov 13;90(5):1907–36. doi: 10.1017/psy.2025.10054 (PMC12805200; doi:10.1017/psy.2025.10054)
Supplement: Furr and Fu supplementary material [file S0033312325100549sup001.zip › SOM_A_Additional_Tables_Model_Comparison.docx]

**Supplemental Online Material A**

**Additional Tables on Model Comparisons in the Simulated Study**

**Table A1**

*Latent Score Recovery of TIRT and Triplet-2PLM on Simulated Triplet Data*

| Slope | Keyed direction | Model | Criterion | Trait 1 | Trait 2 | Trait 3 | Trait 4 | Trait 5 | *M* |
| --- | --- | --- | --- | --- | --- | --- | --- | --- | --- |
| Low | Same | TIRT | MD | -0.01 | -0.01 | 0.00 | 0.00 | 0.00 | 0.00 |
|  |  |  | MAD | 0.53 | 0.52 | 0.53 | 0.53 | 0.52 | 0.53 |
|  |  |  | RMSE | 0.66 | 0.66 | 0.67 | 0.66 | 0.66 | 0.66 |
|  |  |  | MRD | -0.03 | -0.05 | -0.03 | -0.02 | 0.03 | -0.02 |
|  |  |  | Cor | 0.75 | 0.75 | 0.75 | 0.75 | 0.75 | 0.75 |
|  |  | Triplet-  2PLM | MD | -0.01 | -0.01 | 0.00 | 0.00 | 0.01 | 0.00 |
|  |  |  | MAD | 0.53 | 0.53 | 0.53 | 0.53 | 0.53 | 0.53 |
|  |  |  | RMSE | 0.67 | 0.67 | 0.67 | 0.66 | 0.66 | 0.67 |
|  |  |  | MRD | -0.04 | -0.05 | -0.03 | -0.04 | 0.05 | -0.02 |
|  |  |  | Cor | 0.74 | 0.74 | 0.74 | 0.74 | 0.75 | 0.74 |
|  | Mixed | TIRT | MD | -0.01 | -0.01 | 0.00 | 0.00 | 0.00 | 0.00 |
|  |  |  | MAD | 0.42 | 0.43 | 0.44 | 0.43 | 0.44 | 0.43 |
|  |  |  | RMSE | 0.53 | 0.55 | 0.55 | 0.54 | 0.56 | 0.54 |
|  |  |  | MRD | -0.01 | 0.01 | 0.06 | -0.01 | -0.02 | 0.01 |
|  |  |  | Cor | 0.85 | 0.84 | 0.84 | 0.84 | 0.83 | 0.84 |
|  |  | Triplet-  2PLM | MD | -0.01 | -0.02 | 0.00 | 0.00 | 0.01 | -0.01 |
|  |  |  | MAD | 0.42 | 0.44 | 0.44 | 0.43 | 0.45 | 0.44 |
|  |  |  | RMSE | 0.53 | 0.55 | 0.55 | 0.55 | 0.56 | 0.55 |
|  |  |  | MRD | -0.05 | -0.03 | 0.03 | -0.03 | 0.02 | -0.01 |
|  |  |  | Cor | 0.84 | 0.83 | 0.83 | 0.84 | 0.83 | 0.84 |
| Medium | Same | TIRT | MD | 0.01 | -0.01 | 0.01 | -0.01 | 0.00 | 0.00 |
|  |  |  | MAD | 0.49 | 0.47 | 0.48 | 0.47 | 0.48 | 0.48 |
|  |  |  | RMSE | 0.61 | 0.60 | 0.61 | 0.60 | 0.60 | 0.60 |
|  |  |  | MRD | 0.07 | -0.02 | 0.02 | 0.00 | 0.01 | 0.01 |
|  |  |  | Cor | 0.79 | 0.80 | 0.80 | 0.80 | 0.80 | 0.80 |
|  |  | Triplet-  2PLM | MD | 0.00 | 0.00 | 0.00 | -0.01 | 0.01 | 0.00 |
|  |  |  | MAD | 0.49 | 0.48 | 0.48 | 0.48 | 0.48 | 0.48 |
|  |  |  | RMSE | 0.61 | 0.60 | 0.61 | 0.60 | 0.60 | 0.60 |
|  |  |  | MRD | 0.03 | -0.01 | 0.00 | -0.04 | 0.05 | 0.01 |
|  |  |  | Cor | 0.79 | 0.80 | 0.79 | 0.80 | 0.80 | 0.80 |
|  | Mixed | TIRT | MD | 0.01 | -0.01 | 0.01 | 0.00 | 0.00 | 0.00 |
|  |  |  | MAD | 0.31 | 0.31 | 0.31 | 0.31 | 0.31 | 0.31 |
|  |  |  | RMSE | 0.39 | 0.39 | 0.39 | 0.39 | 0.40 | 0.39 |
|  |  |  | MRD | -0.01 | -0.01 | 0.03 | 0.00 | -0.02 | 0.00 |
|  |  |  | Cor | 0.92 | 0.92 | 0.92 | 0.92 | 0.92 | 0.92 |
|  |  | Triplet-  2PLM | MD | 0.00 | -0.01 | 0.00 | -0.02 | 0.01 | 0.00 |
|  |  |  | MAD | 0.31 | 0.31 | 0.31 | 0.31 | 0.32 | 0.31 |
|  |  |  | RMSE | 0.40 | 0.40 | 0.40 | 0.40 | 0.41 | 0.40 |
|  |  |  | MRD | -0.06 | -0.03 | 0.00 | -0.04 | 0.01 | -0.02 |
|  |  |  | Cor | 0.92 | 0.92 | 0.92 | 0.92 | 0.92 | 0.92 |
| High | Same | TIRT | MD | -0.01 | 0.00 | -0.01 | -0.01 | 0.00 | -0.01 |
|  |  |  | MAD | 0.49 | 0.49 | 0.48 | 0.49 | 0.48 | 0.49 |
|  |  |  | RMSE | 0.62 | 0.61 | 0.61 | 0.61 | 0.61 | 0.61 |
|  |  |  | MRD | -0.02 | 0.00 | -0.05 | 0.00 | -0.02 | -0.02 |
|  |  |  | Cor | 0.79 | 0.79 | 0.79 | 0.79 | 0.79 | 0.79 |
|  |  | Triplet-  2PLM | MD | -0.02 | 0.00 | -0.01 | -0.01 | 0.01 | -0.01 |
|  |  |  | MAD | 0.49 | 0.49 | 0.48 | 0.49 | 0.49 | 0.49 |
|  |  |  | RMSE | 0.62 | 0.62 | 0.61 | 0.61 | 0.61 | 0.61 |
|  |  |  | MRD | -0.06 | 0.02 | -0.07 | -0.03 | 0.04 | -0.02 |
|  |  |  | Cor | 0.79 | 0.79 | 0.79 | 0.79 | 0.79 | 0.79 |
|  | Mixed | TIRT | MD | -0.01 | 0.00 | -0.01 | -0.01 | 0.00 | -0.01 |
|  |  |  | MAD | 0.26 | 0.26 | 0.26 | 0.26 | 0.27 | 0.26 |
|  |  |  | RMSE | 0.35 | 0.34 | 0.34 | 0.34 | 0.35 | 0.34 |
|  |  |  | MRD | -0.06 | -0.01 | -0.04 | -0.02 | -0.02 | -0.03 |
|  |  |  | Cor | 0.94 | 0.94 | 0.94 | 0.94 | 0.94 | 0.94 |
|  |  | Triplet-  2PLM | MD | -0.02 | 0.00 | -0.01 | -0.02 | 0.01 | -0.01 |
|  |  |  | MAD | 0.27 | 0.27 | 0.26 | 0.27 | 0.27 | 0.27 |
|  |  |  | RMSE | 0.35 | 0.35 | 0.35 | 0.35 | 0.35 | 0.35 |
|  |  |  | MRD | -0.09 | -0.01 | -0.06 | -0.06 | 0.02 | -0.04 |
|  |  |  | Cor | 0.94 | 0.94 | 0.94 | 0.94 | 0.94 | 0.94 |

*Note*. TIRT was estimated by ULSMV, and Triplet-2PLM was estimated by MML-EM. MD = Mean Deviation; MAD = Mean Absolute Deviation; RMSE = Root Mean Square Error; MRD = Mean Relative Deviation; Cor = Correlation.

**Table A2**

*Comparisons of TIRT, Triplet-2PLM, and MGPPM on Simulated Triplet Data: Running Time (s)*

| Slope | Keyed  direction | TIRT | | Triplet-2PLM | | MGPPM | |
| --- | --- | --- | --- | --- | --- | --- | --- |
|  |  | *M* | *SD* | *M* | *SD* | *M* | *SD* |
| Low | Same | 1.29 | 0.46 | 2927 | 61 | 2373 | 101 |
|  | Mixed | 1.26 | 0.44 | 2409 | 57 | 2367 | 109 |
| Medium | Same | 1.43 | 0.50 | 2487 | 70 | 2480 | 127 |
|  | Mixed | 1.54 | 0.51 | 2599 | 62 | 2489 | 112 |
| High | Same | 2.06 | 0.42 | 3160 | 87 | 2651 | 213 |
|  | Mixed | 1.80 | 0.58 | 3170 | 108 | 2706 | 243 |

*Note.* TIRT was estimated by ULSMV on a laptop with an Intel Core i5-1345U CPU and 16GB RAM, and Triplet-2PLM and MGPPM were estimated by MML-EM on multiple servers in a Linux grid.
